# Impact on child acute malnutrition of integrating small-quantity lipid-based nutrient supplements into community-level screening for acute malnutrition: A cluster-randomized controlled trial in Mali

**DOI:** 10.1371/journal.pmed.1002892

**Published:** 2019-08-27

**Authors:** Lieven Huybregts, Agnes Le Port, Elodie Becquey, Amanda Zongrone, Francisco M. Barba, Rahul Rawat, Jef L. Leroy, Marie T. Ruel

**Affiliations:** Poverty, Health and Nutrition Division, International Food Policy Research Institute, Washington, DC, United States of America; London School of Hygiene and Tropical Medicine, UNITED KINGDOM

## Abstract

**Background:**

Community-based management of acute malnutrition (CMAM) has been widely adopted to treat childhood acute malnutrition (AM), but its effectiveness in program settings is often limited by implementation constraints, low screening coverage, and poor treatment uptake and adherence. This study addresses the problem of low screening coverage by testing the impact of distributing small-quantity lipid-based nutrient supplements (SQ-LNSs) at monthly screenings held by community health volunteers (CHVs). Screening sessions included behavior change communication (BCC) on nutrition, health, and hygiene practices (both study arms) and SQ-LNSs (one study arm). Impact was assessed on AM screening and treatment coverage and on AM incidence and prevalence.

**Methods and findings:**

A two-arm cluster-randomized controlled trial in 48 health center catchment areas in the Bla and San health districts in Mali was conducted from February 2015 to April 2017. In both arms, CHVs led monthly AM screenings in children 6–23 months of age and provided BCC to caregivers. The intervention arm also received a monthly supply of SQ-LNSs to stimulate caregivers’ participation and supplement children’s diet. We used two study designs: i) a repeated cross-sectional study (n = approximately 2,300) with baseline and endline surveys to examine impacts on AM screening and treatment coverage and prevalence (primary study outcomes) and ii) a longitudinal study of children enrolled at 6 months of age (n = 1,132) and followed monthly for 18 months to assess impact on AM screening and treatment coverage and incidence (primary study outcomes). All analyses were done by intent to treat. The intervention significantly increased AM screening coverage (cross-sectional study: +40 percentage points [pp], 95% confidence interval [CI]: 32, 49, *p* < 0.001; longitudinal study: +28 pp, 95% CI: 23, 33, *p* < 0.001). No impact on treatment coverage or AM prevalence was found. Children in the intervention arm, however, were 29% (95% CI: 8, 46; *p* = 0.017) less likely to develop a first AM episode (incidence) and, compared to children in comparison arm, their overall risk of AM (longitudinal prevalence) was 30% (95% CI: 12, 44; *p* = 0.002) lower. The intervention lowered CMAM enrollment by 10 pp (95% CI: 1.9, 18; *p* = 0.016), an unintended negative impact likely due to CHVs handing out preventive SQ-LNSs to caregivers of AM children instead of referring them to the CMAM program. Study limitations were i) the referral of AM cases by our research team (for ethical reasons) during monthly measurements in the longitudinal study might have interfered with usual CMAM activities and ii) the outcomes presented by child age also reflect seasonal variations because of the closed cohort design.

**Conclusions:**

Incorporating SQ-LNSs into monthly community-level AM screenings and BCC sessions was highly effective at improving screening coverage and reducing AM incidence, but it did not improve AM prevalence or treatment coverage. Future evaluation and implementation research on CMAM should carefully assess and tackle the remaining barriers that prevent AM cases from being correctly diagnosed, referred, and adequately treated.

**Trial registration:**

ClinicalTrials.gov NCT02323815.

## Abstract 2

Lieven Huybregts and colleagues show reduced incidence of acute malnutrition in children in Mali when supplements are provided at monthly screening sessions.

## Author summary

**Why was this study done?:**

- Child acute malnutrition (AM) is a serious public health problem affecting 52 million children under the age of 5 years worldwide.
- Many low and middle-income countries have adopted the community-based management of acute malnutrition (CMAM) to treat AM. CMAM is a treatment model endorsed by the World Health Organization that relies on community workers, often volunteers, to conduct first-line screening in the community and refer identified cases to a health facility to enroll in treatment. AM cases without medical complications are treated using energy-dense therapeutic supplements through an outpatient treatment program.
- The effectiveness of CMAM in the field is often limited because of key barriers, including the low proportion of caregivers taking their child to the screening sessions and low enrollment in CMAM treatment.

**What did the researchers do and find?:**

- The researchers randomly allocated 48 health center catchment areas in two health districts in Mali to an intervention arm or a comparison arm. In both study arms, community health volunteers organized monthly meetings at village level to screen children 6–23 months of age for AM and conducted group behavior change communication (BCC) on child nutrition, health, sanitation, and hygiene.
- The intervention arm received small-quantity lipid-based nutrient supplements (SQ-LNSs) to incentivize caregivers to attend these monthly meetings and to increase the intake of energy and essential micronutrients in children. SQ-LNSs consist of a peanut-based spread fortified with vitamins and minerals.
- Researchers found that the intervention led to significantly more children being screened for AM (40 percentage points more than in the comparison arm) but that this result did not lead to more children being treated for AM. The intervention was effective in preventing AM, lowering the risk of developing AM by almost a third.

**What do these results mean?:**

- The SQ-LNSs provided a strong incentive for caregivers to attend the community-level monthly meetings and to get their children screened for AM.
- The lack of impact on AM treatment, despite the positive results on the percentage of children screened, suggests that there remain important barriers that prevent children who are screened and identified as suffering from AM from being referred, enrolled in the program, and treated.
- Providing SQ-LNSs to increase attendance at screening and BCC sessions offered at the community level is an important intervention to prevent child AM. However, more needs to be done to address the constraints that prevent AM children from receiving adequate referral and enrolling and completing CMAM treatment.

## Introduction

Globally, an estimated 52 million children suffer from acute malnutrition (AM) [1]. AM dramatically increases the risk of death; compared to well-nourished children, children with moderate acute malnutrition (MAM) are 3.4 times more likely to die, and this probability increases to a staggering 11.6 times in children with severe acute malnutrition (SAM) [2]. Child AM is the underlying cause for an estimated annual 875,000 deaths of children under 5 years of age, representing about 12.6% of all deaths in this age group worldwide [3].

More than a decade ago, the World Health Organization (WHO) endorsed the community-based management of acute malnutrition (CMAM) model to address SAM. The specific aim was to tackle the low coverage and high case fatality of the existing inpatient treatment model [4]. CMAM entails the active case-finding and referral of children with SAM (and in some settings, also children with MAM) by community health workers (CHWs) or volunteers to first-line health services and the outpatient treatment of children with SAM who demonstrate sufficient appetite and have no medical complications [5]. Two developments facilitated the shift to the outpatient model: first, the introduction of mid-upper arm circumference (MUAC) as an additional diagnostic tool; this change made case detection by CHWs more convenient and affordable than using weight-for-length Z-scores (WLZs), which require the measurement of both weight and length. Second, the introduction of energy-dense ready-to-use therapeutic foods (RUTFs) provided a convenient and safe way to deliver the energy and nutrients needed for children suffering from SAM to recover. The supplements can be consumed directly from the package, do not require a cold chain, and are microbiologically safe [6]. In contexts with a high prevalence of AM, CMAM is often extended to the outpatient treatment of children with MAM using ready-to-use supplementary foods (RUSFs) or fortified flour blends [7,8].

In Mali, AM screening is conducted by community health volunteers (CHVs or *Relais Communautaires* in French), by salaried CHWs (or *Agent de Santé Communautaire*) who typically reside in more remote villages with limited access to health facilities, and by health center staff. CHVs are supervised by both health center staff and by CHWs (in villages where CHWs are active). The national CMAM protocol prescribes active screening and referral for SAM and MAM by CHVs at the community level and passive screening by CHWs and health center staff at every contact with a child [9]. The active screening by CHVs is done through door-to-door household visits or by gathering all caregivers and their children 6–59 months of age at a central location in the community. Using color-coded MUAC tapes, CHVs diagnose children with SAM (MUAC < 115 mm or presence of bilateral pitting edema) or MAM (115 mm ≤ MUAC < 125 mm). Identified SAM and MAM cases are referred to the nearest health center. At the health center, where the SAM diagnosis is confirmed by health center staff if the child presents an MUAC below 115 mm, bilateral pitting edema, or a WLZ < −3, using the WHO child growth standard [10]. MAM is confirmed if the child’s MUAC is between 115 and 125 mm or if the child’s WLZ is between −3 and −2. Children with SAM or MAM who demonstrate sufficient appetite and do not suffer from edema or medical complications are enrolled in the existing outpatient treatment program. This requires caregivers to return to the health center for follow-up consultations with health center staff each week if their child suffers from SAM and fortnightly if they have MAM [9]. At the consultation, children with SAM receive a 7-day supply of RUTFs. In Mali, the national protocol requires children suffering from MAM to receive fortnightly RUSFs or fortified blended food. SAM children with medical complications, edema, or poor appetite, as well as children who do not recover within 3 months through the SAM outpatient treatment program, are referred to second-level health services for inpatient treatment. Children suffering from SAM continue to receive SAM treatment until complete recovery is achieved, i.e., their treatment does not change when children’s status changes to MAM during the recovery process.

Even though CMAM has brought the screening and treatment of AM closer to the community, a review of CMAM programs across more than 20 countries found a wide variation in CMAM treatment coverage ranging from 10% to 60% [9,10]. Key barriers to CMAM treatment are the lack of community awareness and recognition of child AM, the lack of caregiver knowledge of existing CMAM program services, the high opportunity costs of participation for caregivers, the distance to treatment sites, and the stigma around AM and its treatment [9,11–13]. In Mali, a survey listed the low coverage of screening for AM as an important barrier to adequate CMAM treatment coverage [14]. Also in Mali, another study found that over a 4- to 5-month period, only 22% of children were screened by CHVs and 5% by health center staff [15]. These findings stress the need to boost screening coverage and increase caregivers’ knowledge and awareness of AM and of the existing local CMAM services.

The “Innovative Approaches for the Prevention of Childhood Malnutrition” (PROMIS) project implemented by Helen Keller International (HKI) in Mali and Burkina Faso integrated preventive interventions into AM screening to increase screening coverage and subsequently increase CMAM enrollment and reduce AM. This paper reports the main findings from the impact evaluation in Mali, where PROMIS used a community-based intervention platform. Monthly AM screening by CHVs was complemented by behavior change communication (BCC) on nutrition, health, and hygiene practices, and children in the treatment arm also received a monthly supply of small-quantity lipid-based nutrient supplements (SQ-LNSs). We hypothesized that offering monthly SQ-LNSs would increase AM screening coverage and, as a result, AM treatment coverage and reduce the incidence of AM through the BCC and the SQ-LNSs’ contribution to improved complementary feeding practices and nutrient intake. We further hypothesized that the combination of prevention (SQ-LNSs and BCC), early detection, and timely referral and AM treatment would lead to a lower AM prevalence.

The results of the impact evaluation in Burkina Faso are available in a companion paper [16].

## Methods

The study protocol was published previously [17]. We present a summary of the methods here.

### Study context

The study was carried out in Bla and San, two health districts located in the Ségou region of eastern Mali. All 28 health center catchment areas from the Bla health district and 20 (of a total of 30) health center catchment areas from the San health district were eligible for study participation. Prior to randomization, we decided to omit the 10 most southern-adjacent health center catchment areas in San because they were less accessible during the rainy season. Each health district has a district hospital for second-line healthcare. In the Ségou region, 22.2% of children 6–23 months of age suffered from AM in 2016 [18].

### Intervention and theory of change

The intervention was implemented from April 2015 to June 2017 and targeted children 6–23 months of age. Some activities were implemented in both the intervention and comparison arms, whereas others were limited to the intervention arm. The theory of change is summarized in **Box 1**.

**Box 1. Theory of change of the PROMIS intervention**

**Fig 1:**
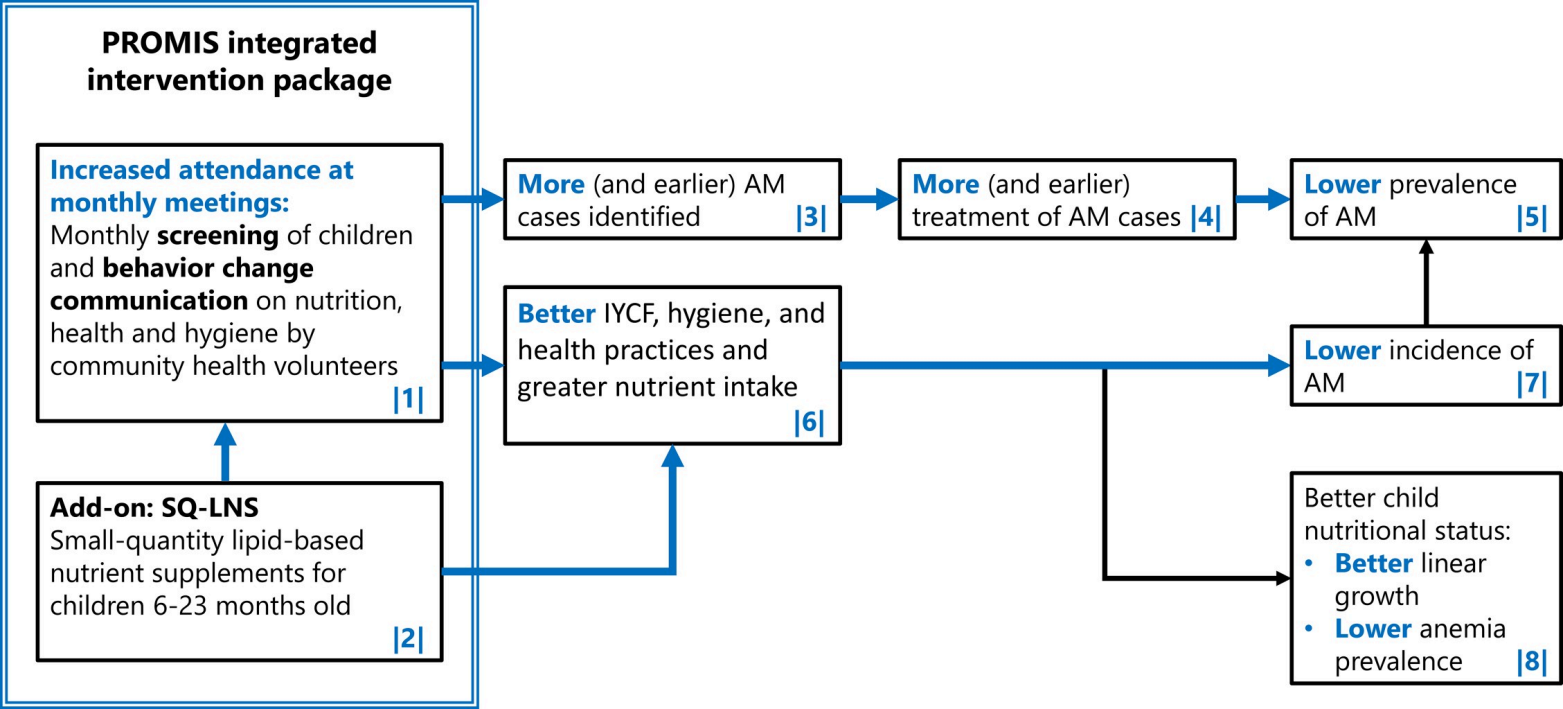
Theory of change of the PROMIS intervention. Hypothesized impacts of the intervention presented in this paper are shown in blue. The blue box represents the intervention. AM, acute malnutrition; IYCF, infant and young child feeding; PROMIS, Innovative Approaches for the Prevention of Childhood Malnutrition; SQ-LNS, small-quantity lipid-based nutrient supplement.

The objective of the PROMIS program was to improve child nutritional status in general and to reduce the incidence and prevalence of AM in particular (**Fig 1**). The program organized monthly meetings between caregivers and CHVs, which provided screening for AM and BCC on nutrition, health, and hygiene (1) and the distribution of monthly doses of SQ-LNS (2). We expected the intervention to impact child AM through two pathways. Along the first pathway (referred to in the text as the “treatment pathway”), increased participation of caregivers in the monthly meetings with CHVs was expected to lead to higher coverage of AM screening (more children being screened more often), which allowed for more AM cases to be identified (3) and for more subsequent referral and treatment (4). In addition, the monthly meetings with CHVs were expected to facilitate the follow-up of previous referrals, which, in turn, was expected to result in better treatment adherence (4). More frequent screening, faster case detection and referral, and better adherence to AM treatment were expected to lead to shorter AM episodes and thus a lower prevalence of AM (5).

Along a second impact pathway (referred to as the “prevention pathway”), frequent caregiver participation in BCC sessions and their children receiving SQ-LNS was expected to lead to better nutrition and health practices (6). As a result, the intervention was expected to lower the incidence of AM (7). Improved nutrition and health practices could also positively impact child linear growth and anemia (8).

A key element in this theory of change is the integration of SQ-LNSs in the monthly AM screening and BCC session delivered by CHVs. Caregivers belonging to the intervention arm received the SQ-LNSs after their child was screened for AM and after they had participated in the BCC. As such, the provision of SQ-LNSs was designed to serve as both an incentive to participation in the screening and BCC sessions and as a nutritional supplement to improve children’s complementary feeding diet.

### PROMIS activities in the intervention and comparison arms

HKI staff trained CHVs to organize monthly village meetings to screen children for AM and provide BCC on nutrition, health, and hygiene practices. At these meetings, CHVs screened all children 6–23 months of age using MUAC and bilateral pitting edema as diagnostic criteria for AM. As per Mali’s AM protocol, CHVs referred MAM and SAM cases to the nearest health center for confirmatory diagnosis and enrollment into the appropriate treatment program.

At each monthly meeting, a BCC session was organized by one or two CHVs in groups of up to 20 caregivers. The BCC content was developed by HKI country teams based on the essential nutrition and hygiene actions framework [19]. Each session covered one topic, which included recommendations on optimal breastfeeding, complementary feeding (quantity, quality, frequency, and preparation), nutrition during pregnancy and lactation, nutrition during child illness and AM, immunization, handwashing, the use of latrines, the installation of a handwashing station, food hygiene, and the use of SQ-LNSs (limited to the intervention arm; see below).

In April 2016, HKI created village nutrition support groups in all study villages consisting of 8 village opinion leaders. In addition to influencing social norms on nutrition, health, and hygiene, the support groups’ activities included mobilizing caregivers to participate in the monthly screening and BCC, supporting the implementation of these activities, and visiting caregivers who did not attend the monthly meeting.

HKI officers supported the CMAM treatment services in all Bla and San health centers by organizing monthly formative supervisions. Besides careful monitoring of stockouts of SAM and MAM treatment products, HKI officers observed the treatment consultations and assessed whether CMAM registers were correctly filled out. The monitoring and evaluation system from HKI did not report any systematic stockouts of SAM and MAM treatment products during the study. At endline, only 5 of 48 health centers reported the occurrence of a stockout of SAM treatment products over the past 6 months. The mean (SD) duration of the stockout was 19 days (13 days) over a period of 6 months. Only two out of 48 health centers reported a stockout of MAM treatment products in the 6 months prior to the endline survey.

### Additional PROMIS activities in the intervention arm

In addition to the activities described above, at each monthly meeting, caregivers in the intervention arm received a monthly supply of thirty 20-g SQ-LNS sachets (Nutriset, Malaunay, France) intended for daily use by the targeted child. SQ-LNSs were only given to children who were diagnosed as not suffering from AM. More information on the development and nutrient composition of SQ-LNSs is given elsewhere [17,20]. Each month, caregivers were instructed by CHVs that the SQ-LNSs were not intended to replace or reduce complementary feeding or breastmilk intake and were encouraged to mix the SQ-LNSs with the child’s complementary foods. Direct consumption from the package was offered as a second option.

The implementation of the PROMIS intervention by CHVs was supervised by either health center staff or CHWs for more remote villages and occasionally by HKI field workers. All CHVs participating in the project received a monthly cash incentive of 2,000 FCFA (approximately US $4) when attending a monthly meeting with HKI staff to discuss program implementation.

Training of health center staff, CHWs, and CHVs on all intervention related activities (screening, BCC, and SQ-LNS distribution) was organized in 2015. For this purpose, existing training guides and manuals were adapted to the Mali context [19,21]. HKI nutrition experts in collaboration with the health district nutrition officer first trained health center staff and CHWs. Subsequently, each health center under supervision of HKI and/or health district officers organized a 3-day training for the CHVs. A total of 1,492 CHVs were trained. A CHV refresher training was organized in 2016. HKI also organized a refresher training for health center staff (2 nurses or nurse assistants per health center) in 2016 on CMAM procedures.

### Study design

We used a two-arm, cluster-randomized, nonblinded effectiveness trial to assess the impact of the integrated preventive PROMIS intervention in children 6–23 months of age. A cluster design was used because individual randomization of the community-based delivery-side intervention was not feasible. A cluster was defined as the catchment area of a health center. All health center catchment areas included in the study were first grouped into strata per health district to account for heterogeneity between health center catchment areas [17]. Health center catchment areas were randomly allocated to the intervention (*n* = 24) or comparison arm (*n* = 24) within each stratum. Randomization was conducted at two public ceremonies in Bla and San, respectively, in the presence of the health center directors and health district authorities, representatives of the communities, and HKI staff who ensured that the study protocol was followed. More details on the randomization procedure are given elsewhere [17].

Two different study designs were used to answer the research questions. A repeated cross-sectional design was used to assess the impact of the intervention after two years of implementation on AM screening coverage, treatment coverage, and AM prevalence. The cross-sectional surveys at baseline (2015) and endline (2017) were conducted in the same calendar months to reduce the effect of seasonality.

The longitudinal study followed a closed cohort of children who were 6–6.9 months of age and not suffering from AM at enrollment. Study children were followed through monthly home visits until they reached 24 months (±1 week) of age. The longitudinal study allowed for the assessment of the impact of the PROMIS intervention on AM incidence, as well as AM screening coverage and treatment coverage. In addition, it provided data on secondary outcomes including the duration of AM episodes, as well as the rate of recovery and relapse.

### Sampling design and sample size calculations

Details on the sampling design can be found elsewhere [17]. For the cross-sectional study, assuming a coefficient of intercluster (i.e., between health center catchment areas) variation k of 0.25, a nonresponse rate of 15%, a type I error of 5%, 80% statistical power, and a baseline AM prevalence of 18.0%. [22], we found that we needed 48 clusters with an average size of 48 children (i.e., an overall sample size of 2,304 children) in each survey to detect a 5.3 percentage point (pp) decrease in AM prevalence. This sample size allowed us to detect a difference in AM screening coverage of 6.7 pp and a difference in AM treatment coverage of 14.7 pp between study arms assuming baseline values of 25% for both outcomes.

For the longitudinal study, we used a coefficient of intercluster variation k of 0.2, a dropout rate of 20%, a type I error of 5%, 80% statistical power, and a baseline incidence of 0.61 case per child-year and found that we needed to recruit 24 children in each of the 48 clusters (i.e., an overall sample size of 1,152 children total) to detect a 23.5% reduction in AM incidence over the 18 months of follow-up. This sample size allows us to detect a difference in AM screening coverage of 4.9 pp and a difference in treatment coverage of 9.6 pp over the 18-month follow-up, assuming baseline values of 25% for both outcomes.

Enumerators conducted a census prior to each cross-sectional survey and prior to the longitudinal study. Study children were randomly sampled from the census list. Study inclusion criteria for the cross-sectional and longitudinal study are shown in **Table 1**.

**Table 1:** Inclusion criteria and primary and secondary study outcomes for the cross-sectional and longitudinal study.

	Cross-Sectional Study	Longitudinal Study
Inclusion criteria		At study enrollment:
	i) being a singleton child 6–23 months (±1 week) of age	i) being a singleton child 6.0–6.9 months of age
	ii) not having congenital malformations that hinder growth and/or anthropometric measurements	ii) not having congenital malformations that hinder growth and/or anthropometric measurements
	iii) child’s principal caregiver having lived in the study area since the child was born	iii) not suffering from AM, with AM defined as WLZ < −2 or MUAC < 125 mm or the presence of bilateral pitting edema
		iv) child’s principal caregiver not planning to leave the study area in the next year
Primary study outcomes	i) AM screening coverage (the number of children screened for AM in the past month over the total number of study children)	i) AM screening coverage (the number of children screened for AM in the past month over the total number of study children considering all monthly visits over the 18-month follow-up)^a^
	ii) AM treatment coverage (number of children with AM under appropriate treatment for their condition (SAM or MAM) in the past month over the total number of AM cases identified at the time of the survey in the study sample)	ii) AM treatment coverage in children enrolled in the CMAM program (the number of AM episodes for which MAM or SAM treatment was received until discharged or recovery over the total number of AM episodes enrolled in a CMAM program over the 18-month follow-up)
	iii) AM prevalence (the number of cases of AM at survey time over the total number of study children)	iii) incidence of the first AM episode over the 18-month follow-up^b^
Secondary study outcomes	Program participation and coverage in the month preceding the survey:	Program participation and coverage over 18 months of follow-up:
	—participation in monthly CHV-led meeting	—participation in CHV-led meetings
		—change in participation in CHV-led meetings with CHVs over time
	—AM screening coverage through the monthly CHV-led meeting^c^	—AM screening coverage through the monthly CHV-led meeting^c^
		—change in AM screening coverage (screening conducted at CHV-led meeting and total screening coverage)^c^
	—total BCC coverage (BCC delivered at CHV-led meeting and through any channel)^c^	—total BCC coverage (BCC delivered at CHV-led meeting and through any channel)^c^
		—change in BCC coverage (BCC delivered at CHV-led meeting and through any channel) over time
	—total SQ-LNS coverage (SQ-LNSs provided at the CHV-led meeting and through any channel)^d^	—total SQ-LNS coverage (SQ-LNSs provided at the CHV-led meeting and through any channel)^d^
		—change in SQ-LNS coverage (SQ-LNSs provided at the CHV-led meeting and through any channel) over time
	AM:	AM:
	—prevalence of MAM (−3 ≤ WLZ < −2 or 115 mm ≤ MUAC < 125 mm)	—longitudinal prevalence of AM (defined as the total time the child was with AM over the total follow-up time)
	—prevalence of SAM (WLZ < −3 or MUAC < 115 mm or presence of bilateral pitting edema)	—longitudinal prevalence of MAM and SAM (total time the child was with MAM or SAM over the total follow-up time respectively)
	—AM status at the time of SQ-LNS distribution, as reported on the PROMIS beneficiary card or by the caregiver in the absence of PROMIS beneficiary card	—change in AM prevalence over time
	—mean WLZ	—change in WLZ over time
	—mean MUAC	—change in MUAC over time
		Treatment enrollment and coverage:
		—AM treatment enrollment and coverage (the number of MAM and SAM episodes in children enrolled in the CMAM program for which MAM- or SAM-appropriate treatment was received)
		—MAM and SAM treatment enrollment and coverage (the number of MAM or SAM episodes in children enrolled in the CMAM program for which MAM- or SAM-appropriate treatment was received, respectively)
		Recovery, relapse, and episode length:
		—recovery of AM, MAM, and SAM after treatment
		—relapse rates of AM, MAM, and SAM
		—mean AM, MAM, and SAM episode length

^a^The monthly measurements done by the research team included anthropometry. When children were identified by the research team as AM, they were referred to the CMAM for ethical reasons. Our measure of screening coverage excludes these measurements because they were not part of the program implementation activities

^b^We limited the analysis of the incidence to the first episode of AM to assess the impact of the preventive components of the intervention without possible interference of treatment of a previous episode. However, to assess the robustness of our findings, we also carried out the analysis using all episodes as a secondary outcome.

^c^Since AM screening and occasionally also BCC were offered by multiple actors (health center consultations, maternity wards, CHWs, and CHVs outside of the project) in the communities, we assessed the impact of the intervention on total AM screening and BCC coverage and specifically through the monthly CHV-led meetings.

^d^Since SQ-LNSs could also have been distributed outside the CHV-led meetings, we assessed total SQ-LNS coverage, which included SQ-LNSs obtained during and outside of the monthly CHV-led meetings.

**Abbreviations:** AM, acute malnutrition; BCC, behavior change communication; CHV, community health volunteer; CHW, community health worker; CMAM, community-based management of acute malnutrition; MAM, moderate acute malnutrition; MUAC, mid-upper arm circumference; SAM, severe acute malnutrition; SQ-LNS, small-quantity lipid-based nutrient supplement; WLZ, weight-for-length Z-score.

### Primary and secondary outcomes

Both the cross-sectional and longitudinal study had three primary outcomes: two related to AM screening and treatment and the third to AM. AM was defined as WLZ < −2 or MUAC < 125 mm or the presence of bilateral pitting edema. The primary and secondary study outcomes for the cross-sectional study and the longitudinal study are shown in **Table 1**.

### Measurements and indicator creation

Research teams independent from the program were recruited to collect the study data. These teams had no role in the implementation of the intervention. There was no exchange of information between study field teams and the local health system or HKI staff. Research teams were blinded to the allocation group of the study clusters.

Questionnaires were administered to CHVs (cross-sectional study only), the head of household, and the main caregiver of the study child (cross-sectional and longitudinal study) through a computer-assisted personal interviewing format built with Surveybe software versions 4 and 5 (Surveybe, Economic Development Initiatives, High Wycombe, UK). Interviews were conducted in the respondents’ language.

Data on participation in the monthly meeting with the CHV, AM screening, BCC, SQ-LNSs, and treatment coverage were collected through caregiver recall. If services (screening, BCC, and SQ-LNSs) were received outside of the monthly meeting with CHVs, caregivers were asked where and from whom they received these services. The age of study children was determined by recording the date of birth from an available birth certificate or a vaccination card (approximately 90% of children). If no records were available, the date of birth was approximated with the aid of a local events calendar (approximately 10% of children).

All anthropometric measurements were taken in duplicate by teams composed of a study anthropometrist and an assistant enumerator. Child length and MUAC were measured twice to the nearest millimeter, and child weight was measured to the nearest 100 g. A third measurement was taken if the difference between the first two measurements was larger than 300 g for weight and larger than 5 mm for length and MUAC. The average of the two (or three) repeated measurements of length, weight, or MUAC was used for the calculation of nutritional status indicators. Anthropometric Z-scores were calculated using the “zscore06” command in Stata, which is based on the 2006 WHO growth standard [23,24]. Anthropometrists and assistants were trained, and their weight, length, and MUAC measurements were standardized against the benchmark measurement of a lead anthropometrist [25] prior to the start of fieldwork and every two months during the longitudinal study thereafter.

For the longitudinal study, an episode of AM was defined as starting from the moment a child was found to be acutely malnourished at the monthly survey visit until the moment the child was free from AM for at least one monthly measurement. MAM and SAM episodes were defined similarly: from the moment a child was found MAM or SAM at the monthly survey visit until the moment the child was free from AM for at least one monthly measurement. Children who suffered from SAM and qualified as MAM during the recovery process were not included in the MAM incidence estimation.

AM relapse was defined as a new episode of AM, MAM, or SAM after an initial episode of AM, MAM, or SAM, respectively. In line with the Mali national CMAM protocol, recovery from AM, MAM, or SAM was defined as achieving a normal nutritional status (defined as MUAC > 125 mm and WLZ > −2 and absence of edema) within 3 months after the initial diagnosis by the study field team. Appropriate nutritional treatment was defined as follows: RUTF (Nutriset) for SAM cases and MAM cases who recovered from SAM; F-75, F-100, or RUTF for inpatient SAM treatment; and RUSF (Nutriset) or any blended flour fortified with micronutrients for MAM cases. RUTF and RUSF are peanut-based spreads that look similar to SQ-LNSs. Each of these three supplements is formulated to address the specific nutritional needs of the groups of children they target.

We used principal component analysis to construct a proxy household wealth index using the cross-sectional baseline survey and the enrollment survey of the longitudinal study (separate indices were created for the cross-sectional and longitudinal study). The following variables were included: ownership of various assets (each asset included if owned by between 5% and 95% of all households), housing materials, primary source of lighting, primary energy source, and home ownership. Tertiles of the first principal component (i.e., the one with the highest eigenvalue) were used in all analyses [26].

### Statistical analysis

For the descriptive analyses, we calculated proportions, means, and SDs by study arm. A critical *p*-value of 0.025 was calculated using the Benjamini–Hochberg method, assuming a false discovery rate of 5% to account for the potential false discovery rate associated with conducting hypothesis tests on 6 primary study outcomes [27]. Statistical significance for all other analyses was set at 0.05, and all tests were two-sided. Statistical analyses were conducted using Stata 15.0 (Statacorp, College Station, TX, USA). To assess the robustness of our findings, we repeated all regression analyses adjusting for covariates that appeared unbalanced at baseline.

### Cross-sectional study

Linear and linear probability mixed-effects regression models were used to assess the impact of the intervention on continuous and binary outcomes measured at endline, respectively. In case of linear probability regression models, we used robust estimation of standard errors to account for heteroscedasticity of residuals. These regression models included health center catchment area as a random intercept and health district and health center stratum as fixed effects to account for the stratified clustered sampling design. Regression models were adjusted for the cluster baseline means of the outcome (where possible), child age and sex, and whether the child was the first liveborn.

### Longitudinal study

Changes over time in participation in the monthly CHV meeting, AM screening coverage, BCC coverage, SQ-LNS coverage, child MUAC, WLZ, and AM prevalence were modeled using mixed-effects models with restricted cubic splines with health center catchment area and child as random effects to account for clustering. Restricted cubic splines were chosen to model the nonlinear association between these outcomes and child age. To determine the number and location of the knots, we plotted the first derivative of a kernel-weighted local polynomial function against child age. Local maxima and minima appeared at approximately 9, 12, and 16 months for the anthropometric outcomes and at 9, 15, and 22 months for all other outcomes. To assess the robustness of our choice of knots, we repeated our analysis placing knots at each quartile of child age [28]. Akaike and Bayesian information criteria of both models proved to be very similar (<5% difference). We evaluated the impact of the intervention on changes in the aforementioned outcomes over time by testing the intervention × cubic age splines interactions jointly using a Wald chunk test.

We plotted the Kaplan–Meier failure function to visualize the impact of the intervention on the incidence of a child’s first episode of AM. The impact of the intervention on AM, MAM, and SAM incidence, relapse rate, and longitudinal prevalence was analyzed using mixed-effects Poisson regression models with robust estimation of standard errors and health center catchment area as random intercepts. The Poisson model was used to estimate adjusted incidence rate ratios and, in the case of longitudinal prevalence, risk ratios rather than odds ratios, as recommended for prospective studies [29–31]. We used linear probability mixed-effects regression models to assess the impact of the intervention on binary AM treatment outcomes with robust estimation of standard errors and a linear mixed-effects regression model in order to assess the impact of the intervention on episode length with health center and child as random intercepts.

All longitudinal regression models were adjusted for the calendar month of enrollment, health district, the health center sampling stratum, child age and sex, the baseline value of the outcome where possible, and whether the child was the first liveborn.

All analyses were done using a full intent-to-treat approach to reduce potential bias arising from missing data. Therefore, prior to analysis, we conducted multiple imputations of missing longitudinal continuous and binary outcome data using a 2-fold fully conditional specification (FCS) algorithm [32,33]. This algorithm is an extension of the standard FCS [34] developed for a longitudinal study design with repeated measurements. Contrary to standard FCS, in which missing data are imputed from the distribution of observations at a given time point conditional on a set of covariates, the 2-fold FCS imputes missing values under the missing at random assumption at a given time point. It does so by using a model with covariate information from that time point and from adjacent time points, respecting the temporal ordering of observations. The information on which we conditioned the imputed values consisted of health center catchment area, health center stratum, health district, child sex, whether the child was the first liveborn of the caregiver, child age, and maternal height. All longitudinal analyses were conducted on imputed data generated by 50 iterations using the “mi estimate” commands in Stata.

### Study registration and ethics

The trial was registered on December 18th, 2014 (prior to study enrollment) with clinicaltrials.gov under identifier NCT02323815, and the study protocol was published [17]. The study protocol was approved by the ethics committee of the Faculty of Medicine, Pharmacy and Onto-Stomatology (FMPOS) of the University of Bamako (#2014/110/CE/FMPOS) and the institutional review board at the International Food Policy Research Institute (IRB #00007490). Prior to inclusion in the baseline or endline surveys or the longitudinal study, information about the study was given orally and in writing to caregivers of potentially eligible children, and informed consent was documented through signature or fingerprint for illiterate caregivers. If, at one of the enumerator home visits, a child was found to be suffering from AM, severe anemia (cross-sectional study only; measured by portable Hemocue 201+ device [Hemocue, Ängelholm, Sweden]), malaria (longitudinal study only; diagnosed using a fingerpick rapid diagnostic test in cases in which the child’s axillary temperature was over 37.5°C or the mother reported fever in the last 24 h), or demonstrated any general danger sign (altered consciousness, repeated vomiting, refusal to eat and drink, convulsions) that required immediate medical care, enumerators informed their supervisor, who filled out a referral slip to the nearest health center for treatment. CMAM and malaria treatment were offered free of charge at the health center. For other types of referrals, the project reimbursed health centers for costs incurred. This study is reported as per the Consolidated Standards of Reporting Trials (CONSORT) guideline (see S1 CONSORT checklist).

## Results

### Cross-sectional study

#### Participants’ trial profile and characteristics

**Fig 2:**
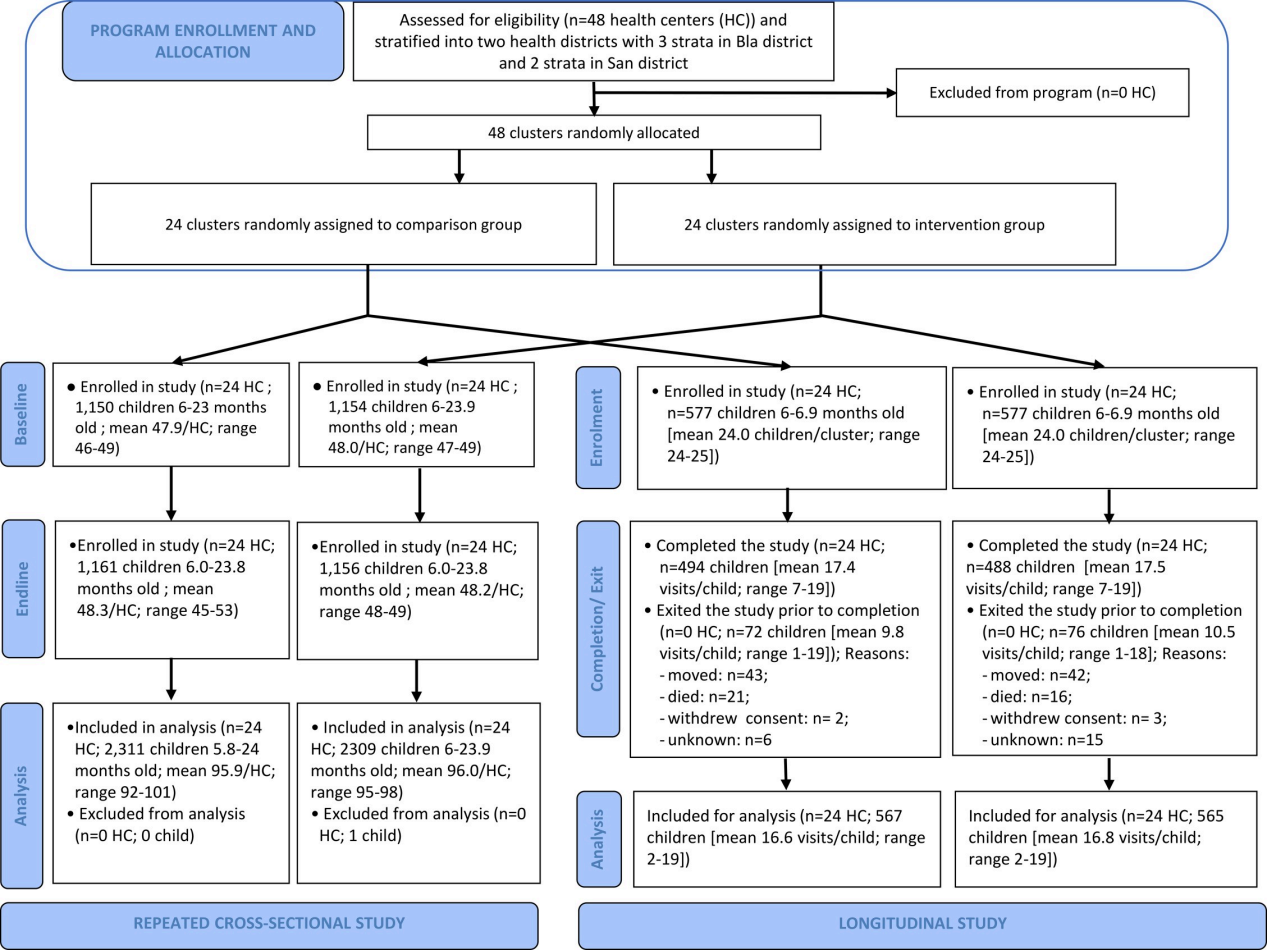
Trial profile for repeated cross-sectional study and longitudinal study. HC, health center.

Data from all 48 study clusters were used for the analysis of the cross-sectional study (Fig 2). The cross-sectional study included 2,304 children from 2,196 households in the baseline survey (February–March 2015) and 2,316 children from 2,195 households in the endline survey (February–March 2017).

Roughly a third of households in both study arms were food insecure **(Table 2)**. Schooling was low, with only 25% of heads of households and 7% of caregivers having completed primary education. Almost all caregivers (97%) reported having initiated breastfeeding within an hour after delivery. Infant and young child feeding (IYCF) practices were suboptimal, with only about a quarter of study children being fed a minimum acceptable diet. A very high proportion of study children suffered from anemia (83%). Overall, baseline characteristics were comparable between study arms, except for the mean linear distance to the nearest health center (shorter in the intervention arm), use of an improved water source (higher in the intervention arm), presence of improved sanitation facility (higher in the intervention arm), and the proportion of children being fed meals with a minimum meal frequency (higher in the comparison arm).

**Table 2:** Baseline (cross-sectional study) and enrollment (longitudinal study) sample characteristics by study arm.

		Cross-Sectional Study		Longitudinal Study	
		Comparison	Intervention	Comparison	Intervention
**Cluster characteristics**		n = 24	n = 24	n = 24	n = 24
Number of villages per cluster		2.8 ± 0.8	3.0 ± 0.9	2.7 ± 0.7^a^	3.0 ± 0.9
**Household characteristics**		n = 1,092	n = 1,104	n = 567	n = 565
Linear distance from household to health center, km^b^		4.7 ± 4.5	4.0 ± 3.7	4.4 ± 4.4	3.4 ± 3.4
Household size		7.0 ± 3.2	6.8 ± 2.9	6.2 ± 3.3	5.8 ± 2.8
Relative wealth status					
	Low	363 (33%)	369 (33%)	194 (34%)	183 (34%)
	Average	343 (31%)	389 (35%)	170 (30%)	208 (37%)
	High	386 (35%)	346 (31%)	203 (36%)	174 (31%)
Household food insecurity^c^		384 (35%)	378 (34%)	187 (33%)	177 (31%)
Water and sanitation^d^					
	Improved primary water source	545 (50%)	723 (66%)	279 (51%)	279 (51%)
	Improved sanitation facility	506 (46%)	623 (56%)	397 (72%)	423 (77%)
**Head of household characteristics**		n = 1,092	n = 1,104	n = 567	n = 565
Age, years		39 ± 10	39 ± 9.9	38 ± 10.0	38 ± 9.2
Male		1,078 (99%)	1,081 (98%)	565 (99%)	561 (99%)
Completed primary education		246 (23%)	301 (27%)	60 (11%)	64 (11%)
**Main caregiver characteristics**		n = 1,148	n = 1,154	n = 567	n = 565
Age, years		29 ± 8.1	28.9 ± 7.4	27.9 ± 6.8	27.7 ± 6.7
Married living with spouse		1,023 (89%)	1,041 (90%)	507 (89.4%)	506 (90%)
Completed at least primary education		75 (6.5%)	92 (8.0%)	43 (7.6%)	50 (8.9%)
Number of food groups consumed^e^		4.5 ± 1.4	4.4 ± 1.3	3.7 ± 1.1	3.6 ± 1.1
Minimum dietary diversity^f^		559 (49%)	527 (46%)	128 (23%)	102 (18%)
**Child characteristics**		n = 1,150	n = 1,154	n = 567	n = 565
Age, months		15 ± 5.1	15 ± 5.2	6.5 ± 0.4	6.5 ± 0.3
Male		591 (51%)	564 (49%)	295 (52%)	294 (52%)
First liveborn		167 (15%)	148 (13%)	87 (15%)	97 (17%)
Initiation of breastfeeding within 24 h^g^		1,111 (97%)	1,113 (96%)		
Timely introduction of (semi)solid and soft foods^h^		75 (48%)	82 (48%)		
Minimum dietary diversity^i^		531 (46%)	495 (43%)		
Minimum meal frequency^j^		739 (64%)	655 (57%)		
Minimum acceptable diet^k^		335 (29%)	304 (26%)		
Consumption of iron-rich or iron-fortified foods^l^		641 (56%)	637 (55%)		
Anemic^m^		958 (83%)	948 (82%)		

Data are mean ± SD or n (%)

^a^Two smaller villages that were included in the cross-sectional study were omitted from the comparison arm because enumerator teams could not find eligible children 6–6.9 months old.

^b^Linear distance between households and the nearest health center was calculated using Global Position System coordinates collected at household and health center level. Data only available for n = 1,005 households in the comparison arm and n = 1,067 households in the intervention arm.

^c^Assessed by FANTA/USAID’s Household Food Insecurity Access Scale [35].

^d^Protected well, borehole, pipe, and rain were considered improved water sources, and improved sanitation facility consisted of pit latrine with slab.

^e^Out of a maximum of 10 food groups as proposed by [36], these being starchy staple foods, nuts and seeds, flesh foods, dark green leafy vegetables, pulses, dairy, eggs, vitamin-A–rich fruits and vegetables, other vegetables, and other fruits.

^f^Minimum dietary diversity for women indicator defined by a consumption of minimally 5 out of 10 food groups over the past 24-h as proposed by FAO technical group [36].

^g^Child breastfed within 24 h after delivery.

^h^Introduction of (semi)solid or soft foods over the past 24 h, WHO-IYCF indicator measured in subsamples of n = 156 and n = 171 6–8 months old in comparison and intervention arm, respectively [37].

^i^Consumed at least 4 food groups in the past 24 h out of the following 7: grains, roots, and tubers; legumes and nuts; dairy products; flesh foods; eggs; vitamin-A–rich fruit and vegetables; other fruits and vegetables [37].

^j^Minimum meal frequency as appropriate for age and breastfeeding status [37].

^k^Composite indicator that combines achievement of the minimum dietary diversity and age-appropriate minimum meal frequency[37].

^l^Defined by consumption of flesh foods or food fortified with iron over past 24 h [37].

^m^Anemia defined as hemoglobin concentration below 11 g.dl^−1^,hemoglobin concentration measured by Hemocue 201+ device (Hemocue).

**Abbreviations:** FANTA, Food and Nutrition Technical Assistance III Project; FAO, Food and Agriculture Organization; IYCF, infant and young child feeding; USAID, United States Agency for International Development; WHO, World Health Organization.

#### Impact on coverage of AM screening and preventive services

The intervention resulted in a 40 pp (95% CI: 32, 49; *p* < 0.001) higher AM screening coverage (primary outcome) at endline **(Table 3)**. In addition, the intervention had a significant positive impact on the coverage of program-specific services: total BCC coverage was 42 pp higher in the intervention arm (95% CI: 33, 51; *p* < 0.001), and total SQ-LNS coverage was 73 pp higher (95% CI: 67, 78; *p* < 0.001) than in the comparison arm. Attendance at the monthly meeting with CHVs was 41 pp (95% CI: 30, 52; *p* < 0.001) higher in the intervention compared to the comparison arm. The program also had a positive impact on the coverage of program-specific services offered through the dedicated PROMIS platform (i.e., the monthly CHV meeting): impact estimates were +33 pp (95% CI: 22, 44; *p* < 0.001) for AM screening coverage, +38 pp (95% CI: 27, 48; *p* < 0.001) for BCC coverage, and +47 pp (95% CI: 36, 57; *p* < 0.001) for SQ-LNS coverage. Among the group of caregivers who received SQ-LNSs outside of the CHV meeting, 58% obtained it at the CHV’s domicile.

**Table 3:** Effect of intervention on coverage of AM screening, BCC, and SQ-LNSs in the past month, assessed by cross-sectional and longitudinal study.

	Cross-Sectional Study (Endline)					Longitudinal Study				
	Comparison	Intervention	Δ^a^ (pp)	95% CI	*p*-Value	Comparison	Intervention	Δ^b^ (pp)	95% CI	*p*-Value
	n = 1,161^c^	n = 1,155^c^				n = 9,424^d^	n = 9,434^d^			
AM screening coverage (primary outcome)	264 (23%)	728 (63%)	40	(32, 49)	<0.001*	888 (9.3%)	3,557 (38%)	28	(23, 33)	<0.001*
AM screening coverage through monthly meeting^e^	113 (9.7%)	489 (42%)	33	(22, 44)	<0.001	243 (2.5%)	2,220 (24%)	21	(16, 27)	<0.001
BCC coverage	191 (17%)	674 (58%)	42	(33, 51)	<0.001	473 (4.9%)	2,861 (30%)	26	(20, 31)	<0.001
BCC coverage through monthly meeting^e^	155 (13%)	588 (51%)	38	(27, 48)	<0.001	390 (4.1%)	2,814 (30%)	26	(20, 32)	<0.001
SQ-LNS coverage	10 (0.86%)	845 (73%)	73	(67, 78)	<0.001	86 (0.91%)	6,475 (60%)	66	(61, 70)	<0.001
SQ-LNS coverage through monthly meeting^e^	1 (0.09%)	539 (47%)	47	(36, 57)	<0.001	31 (0.32%)	2,768 (29%)	29	(23, 36)	<0.001
Participation in monthly meeting	157 (14%)	627 (54%)	41	(30, 52)	<0.001	403 (4.2%)	3,213 (34%)	30	(23, 36)	<0.001

Data are n (%) unless specified otherwise.

*Statistically significant after correcting for multiple testing of primary outcomes, using a *p*_critical_ = 0.025 calculated using the Benjamini–Hochberg method. ICC for primary outcomes are presented in S2 Table.

^a^Difference between intervention and comparison arm in pp, analyzed using a mixed-effects linear probability regression model with health center catchment area as random effect and sampling strata, health district, child sex, whether the child was the first liveborn, child age, and intervention as fixed effects.

^b^Difference between intervention and comparison arm in pp, analyzed using a mixed-effects regression model with restricted cubic splines. Knots were set at 9, 15, and 22 months. Regression models were adjusted for health center catchment area and child as random effects and sampling strata, health district, month of inclusion, child sex, whether the child was the first liveborn, age splines, and intervention as fixed effects.

^c^Number of study children.

^d^Number of child visits.

^e^Service received specifically during the monthly meeting between caregivers and CHVs.

**Abbreviations:** AM, acute malnutrition; BCC, behavior change communication; CHV, community health volunteer; CI, confidence interval; ICC, intracluster correlation coefficient; pp, percentage point; SQ-LNS, small-quantity lipid-based nutrient supplement.

#### Impact on AM treatment

The intervention had no impact on treatment coverage for AM (primary outcome) at endline (Table 4). Only 7.6% of AM cases in the two study arms combined received appropriate treatment in the month preceding the endline survey. An unexpected finding was that 54% of caregivers of children with AM reported that they had received SQ-LNSs in the month preceding the survey, with 33% of caregivers of children with AM having received the SQ-LNSs through the monthly meeting with the CHV.

**Table 4:** Effect of intervention on AM treatment coverage assessed by cross-sectional study.

		Baseline		Endline				
		Comparison	Intervention	Comparison	Intervention	Δ (pp)^a^	95% CI	*p*-Value
**Children with AM at the time of the survey**		n = 172	n = 188	n = 169	n = 156			
	Treatment coverage (primary outcome)^b^	30 (17%)	23 (12%)	17 (10%)	8 (5.1%)	−4.3	(−10, 1.5)	0.14*
	Received any type of AM treatment product^c^	31 (18%)	24 (13%)	17 (10%)	10 (6.4%)	−3.4	(−9.1, 2.2)	0.24
**Children with MAM at the time of the survey**		n = 141	n = 155	n = 128	n = 123			
	Treatment coverage^b^	26 (18%)	19 (12%)	14 (11%)	7 (5.7%)	−4.9	(−12, 2.3)	0.19
	Received a MAM treatment product	17 (12%)	7 (4.5%)	12 (9.4%)	5 (4.1%)	−4.6	(−11, 2.0)	0.18
	Received a SAM treatment product	14 (9.9%)	16 (10%)	4 (3.1%)	2 (1.6%)	−2.3	(−6.7, 2.2)	0.32
**Children with SAM at the time of the survey**		n = 31	n = 33	n = 41	n = 33			
	Treatment coverage^b^	4 (13%)	4 (12%)	3 (7.3%)	1 (3.0%)	−4.7	(−15, 5.6)	0.37
	Received any type of AM treatment product^c^	5 (16%)	5 (15%)	3 (7.3%)	3 (9.1%)	−2.1	(−12, 7.9)	0.67

Data are n(%) or mean ± SD. **Abbreviations:** AM, acute malnutrition; CI, confidence interval; ICC, intracluster correlation coefficient; MAM, moderate acute malnutrition; pp, percentage point; SAM, severe acute malnutrition.

*Not statistically significant after correcting for multiple testing of primary outcomes, using a *p*_critical_ = 0.025 calculated using the Benjamini–Hochberg method. ICC for primary outcomes are presented in **S2 Table**.

^a^Difference between intervention and comparison arm expressed in percentage point analyzed using a mixed-effect linear probability regression model with health center as random effect and sampling strata, health district, child sex, whether the child was the first liveborn, child age, the cluster means of the outcome at baseline, and intervention as fixed effects.

^b^Treatment coverage is defined by MAM children receiving a MAM or SAM treatment product and SAM children receiving a SAM treatment product in the past month.

^c^Any type of AM treatment product refers to MAM and SAM treatment products used by the health services.

#### Impact on child AM

We did not find any impact of the intervention on the prevalence of AM (primary outcome) or the prevalence of SAM or MAM (secondary outcomes) (**Table 5**). Mean WLZ (secondary outcome) in the intervention arm, however, was 0.10 (95% CI: 0.01, 0.20; *p* = 0.034) units higher than in the comparison arm at endline. There was no impact on MUAC.

**Table 5:** Effect of intervention on AM outcomes assessed by cross-sectional study.

	Baseline		Endline				
	Comparison	Intervention	Comparison	Intervention	Δ	95% CI	*p*-Value
	n = 1,148	n = 1,153	n = 1,159	n = 1,154			
AM prevalence (primary outcome)	172 (15%)	188 (16%)	169 (15%)	156 (14%)	−1.31^a^	(−4.2, 1.6)	0.37*
MAM prevalence	141 (12%)	155 (13%)	128 (11%)	123 (11%)	−0.43^a^	(−3.0, 2.1)	0.75
SAM prevalence	31 (2.7%)	33 (2.9%)	41 (3.5%)	33 (2.9%)	−0.79^a^	(−2.3, 0.76)	0.30
WLZ	−0.78 ± 1.00	−0.74 ± 1.00	−0.73 ± 1.01	−0.61 ± 0.98	0.10^b^	(0.01, 0.20)	0.034
MUAC, mm	138 ± 11	138 ± 11	137 ± 11	138 ± 11	0.92^b^	(−0.18, 2.02)	0.10

Data are n(%) or mean ± SD. AM, acute malnutrition; CI, confidence interval; ICC, intracluster correlation coefficient; MAM, moderate acute malnutrition; MUAC, mid-upper arm circumference; SAM, severe acute malnutrition; WLZ, weight-for-length Z-score.

*Not statistically significant after correcting for multiple testing of primary outcomes, using a *p*_critical_ = 0.025 calculated using the Benjamini–Hochberg method. ICC for primary outcomes are presented in **S2 Table**.

^a^Difference between intervention and comparison arm expressed in percentage points analyzed using a mixed-effect linear probability model with health center as random effect and sampling strata, health district, child sex, child age and whether the child was the first liveborn, the cluster means of the outcome at baseline, and intervention as fixed effects.

^b^Difference between intervention and comparison analyzed using a linear mixed- model with health center as random effect and sampling strata, health district, child sex, child age, whether the child was the first liveborn, cluster means of the outcome at baseline, and intervention as fixed effects.

### Longitudinal study

#### Participants’ trial profile and characteristics at enrollment

None of the 48 study clusters were lost to follow-up (**Fig 2**). The longitudinal study enrolled 1,132 children from 1,132 households between July and October 2015. The last follow-up visits were conducted in April 2017. A total of 150 (13%) children were lost to follow-up with similar attrition patterns for the intervention and control group. Most of the attrition (n = 75) happened during the last 6 months of follow-up. The main reasons for attrition were caregiver–child dyads who moved away from the study districts (n = 86/150) and child death (n = 37/150). Sample characteristics at enrollment were similar between study arms (**Table 2**), except for the distance to the nearest health center (shorter in the intervention arm) and the presence of an improved sanitation facility (more likely in the intervention arm). Over 18 months of follow-up, we observed 21 child deaths in the comparison group and 16 child deaths in the intervention group **(Fig 2)**.

#### Impact on coverage of AM screening and preventive services

The intervention led to a higher AM screening coverage (primary outcome) (+28 pp; 95% CI: 23, 34; *p* < 0.001) in the intervention versus comparison arm over the 18-month study period (Table 3). The intervention also had a significant positive impact on BCC and SQ-LNS coverage of 26 pp (95% CI: 20, 31; *p* < 0.001) and 66 pp (95% CI: 61, 70; *p* < 0.001), respectively. Attendance at the monthly meeting with CHVs was 30 pp (95% CI: 23, 36; *p* < 0.001) higher in the intervention arm than in the comparison arm. The intervention also had a positive impact on intervention coverage of different interventions at CHV meetings: the impact on AM screening was +21 pp (95% CI: 16, 27; *p* < 0.001), on BCC coverage was +26 pp (95% CI: 20, 32; *p* < 0.001), and on SQ-LNS coverage was +29 pp (95% CI: 23, 36; *p* < 0.001).

**Fig 3:**
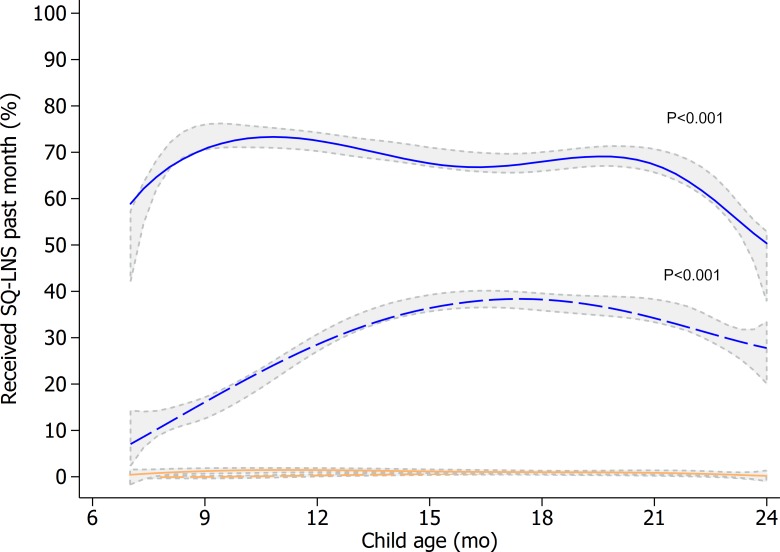
Total SQ-LNS coverage and SQ-LNS coverage through the meeting with CHVs in the longitudinal study by child age and by study arm. Line graphs represent the fitted values of the proportion of children who received SQ-LNS in the intervention arm (blue) and the comparison arm (orange) through the monthly meeting with CHV (dashed lines) or from any source (solid line). Gray areas represent 95% confidence bands of kernel-weighted local polynomial smoothed values by study arm using the observed data. Analysis is based on n = 9,424 child visits in the comparison arm and n = 9,434 in the intervention arm. Mixed-effects regression models with restricted cubic splines (knots at 9, 15, and 22 months of child age) were used with health center catchment area and child as random intercepts and health district, sampling strata, month of inclusion, child sex, whether the child was a first live birth or not, age splines, and intervention as fixed effects. A chunk Wald test was used to test the “age spline × intervention” interaction terms (*p*-values shown). CHV, community health volunteer; SQ-LNS, small-quantity lipid-based nutrient supplement.

From the start of the program, a large and consistent proportion of study caregivers (60%–70%) reported having received SQ-LNSs (**Fig 3**). SQ-LNSs received through the monthly meeting with CHVs, however, was below 10% at program start and increased to only 30% to 40% over time. Monthly attendance at the CHV-led meetings, the AM screening, and the BCC sessions steadily increased with child age and plateaued at around 15 months of age (**S1 Fig, S2 Fig and S3 Fig**).

#### Impact on AM treatment

The intervention had no impact on AM treatment coverage (primary outcome) or on the secondary outcomes of AM treatment initiation, AM recovery rate, or episode length (Table 6). In fact, the program had a negative impact of 10 pp (95% CI: −18, −1.9; *p* = 0.016) on the number of AM episodes of children enrolled in the CMAM program. Similar results were found for MAM (statistically significant) and SAM (marginally significant). Over the 18-month follow-up, 53% of caregivers of AM children reported that they had received SQ-LNSs in the month preceding the survey (contrary to protocol), with 19% of them having received the SQ-LNSs at the monthly meeting with the CHV. Among the subgroup of children with AM who received SQ-LNSs, up to 86% did not receive AM treatment.

**Table 6:** Effect of the intervention on CMAM enrollment, treatment, and recovery outcomes for AM episodes assessed by longitudinal study.

	Comparison	Intervention	Δ^a^	95% CI	*p*-Value
**AM episodes**	n = 595	n = 452			
Enrolled in CMAM program	265 (45%)	154 (34%)	−10	(−18, −1.9)	0.016
	n = 265	n = 154			
Treatment coverage (primary outcome)^b^	128 (48%)	75 (49%)	0.90	(−9.2, 11)	0.86*
Treatment initiated^c^	223 (84%)	130 (85%)	1.9	(−7.0, 11)	0.67
Recovery within 3 months after enrollment	219 (83%)	130 (85%)	2.2	(−5.2, 9.6)	0.56
Length of enrolled episodes, d^d^	74 ± 67	66 ± 50	−7.6^e^	(−22, 6.4)	0.29
**MAM episodes**	n = 508	n = 389			
Enrolled in CMAM program	226 (45%)	137 (35%)	−8.3	(−16, −0.10)	0.047
	n = 226	n = 137			
Treatment coverage^b^	96 (42%)	53 (39%)	−1.6	(−11, 8.0)	0.74
Treatment initiated^c^	161 (71%)	96 (70%)	0.37	(−11, 12)	0.95
Recovery within 3 months after enrollment	199 (88%)	121 (88%)	−0.76	(−8.7, 7.1)	0.85
Length of enrolled episodes, d^d^	60 ± 47	60 ± 41	0.83 ^e^	(−10, 12)	0.88
**SAM episodes**	n = 120	n = 78			
Enrolled in CMAM program	58 (48%)	25 (32%)	−15	(−32, 1.2)	0.07
	n = 58	n = 25			
Treatment coverage^b^	5 (8.1%)	6 (24%)	15	(−3.4, 34)	0.11
Treatment initiated^c^	23 (40%)	12 (50%)	11	(−14, 36)	0.39
Recovery within 3 months after enrollment	39 (67%)	17 (67%)	0.56	(−19, 20)	0.96
Length of enrolled episodes, d^d^	98 ± 90	82 ± 66	−5.5^e^	(−48, 37)	0.80

Data are n(%) or mean ± SD. **Abbreviations:** AM, acute malnutrition; CI, confidence interval; CMAM, community-based management of acute malnutrition; ICC, intracluster correlation coefficient; MAM, moderate acute malnutrition; pp, percentage point; SAM, severe acute malnutrition.

*Not statistically significant after correcting for multiple testing of primary outcomes, using a *p*_critical_ = 0.025 calculated using the Benjamini–Hochberg method. ICC for primary outcomes are presented in **S2 Table**.

^a^Difference between intervention and comparison arm expressed in pp (unless specified otherwise), analyzed using a mixed-effect linear probability regression model with health center and child as random effects and sampling strata, health district, month of inclusion, child sex, whether the child was the first liveborn, child age at the start of an episode, and intervention as fixed effects, unless specified otherwise.

^b^Treatment coverage defined as the proportion of AM, MAM, SAM children that received continuous treatment from CMAM enrollment onwards over the total number of enrolled AM, MAM, SAM children, respectively.

^c^Treatment initiated implies that AM children received either a MAM or SAM treatment, MAM children received MAM treatment, and SAM children received SAM treatment.

^d^Episode length is measured from the onset of the AM, MAM, or SAM episode until the moment the child is free from AM for at least one monthly measurement.

^e^Difference in mean episode length (days) between intervention and comparison arm analyzed using a linear mixed-effects regression model with health center and child as random effects and sampling strata, health district, month of inclusion, child sex, whether the child was the first liveborn, child age at the start of an episode, and intervention as fixed effects.

#### Impact on child AM

**Fig 4:**
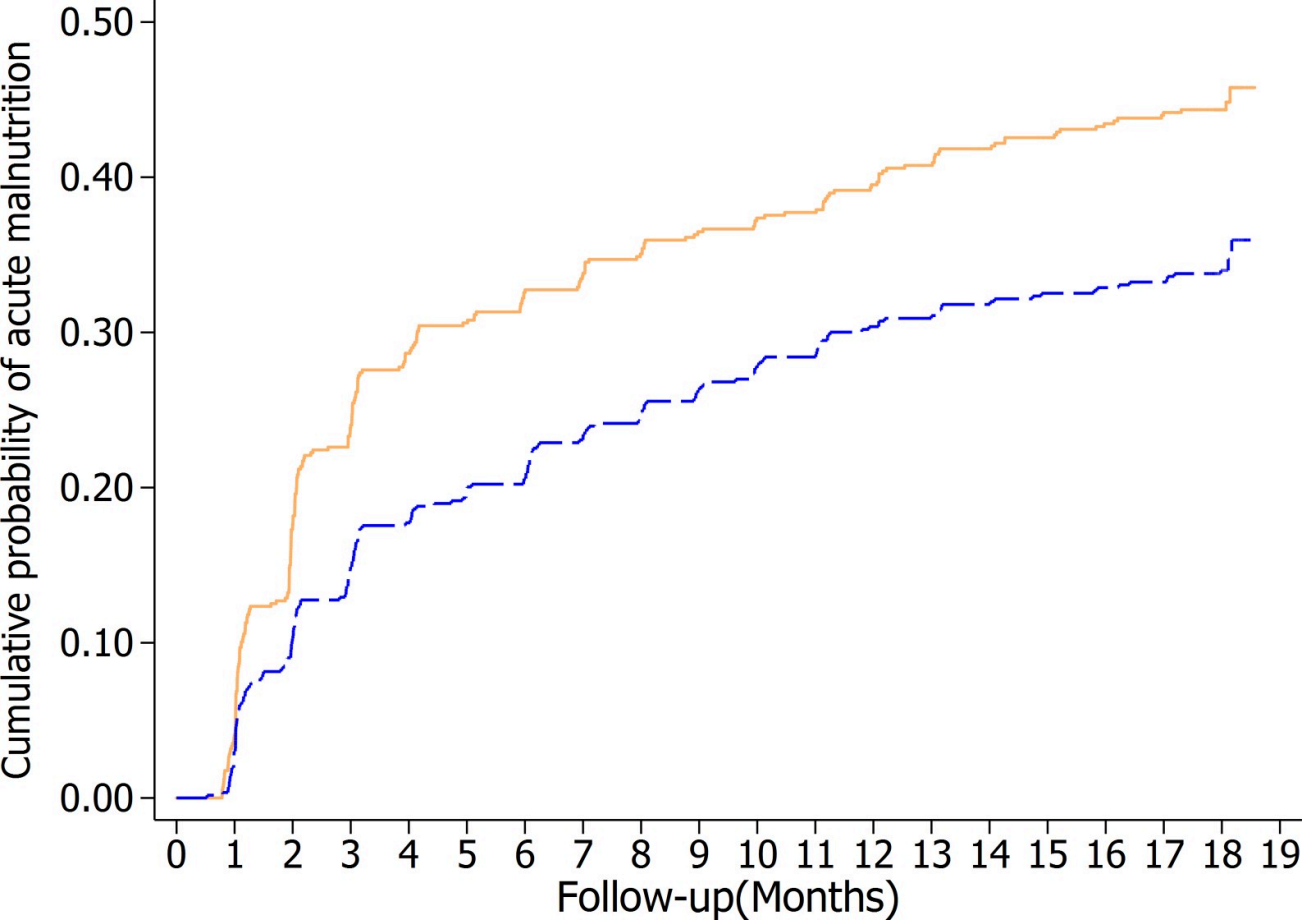
Kaplan–Meier failure plot showing the cumulative probability of child AM by study arm using the longitudinal study data (n = 567 children in the comparison arm contributing 535 child-years of follow-up; n = 565 in the intervention arm contributing 604 child-years of follow-up). The blue dashed line represents results from the intervention arm, and the orange solid line represents results from the comparison arm. AM, acute malnutrition.

**Fig 5:**
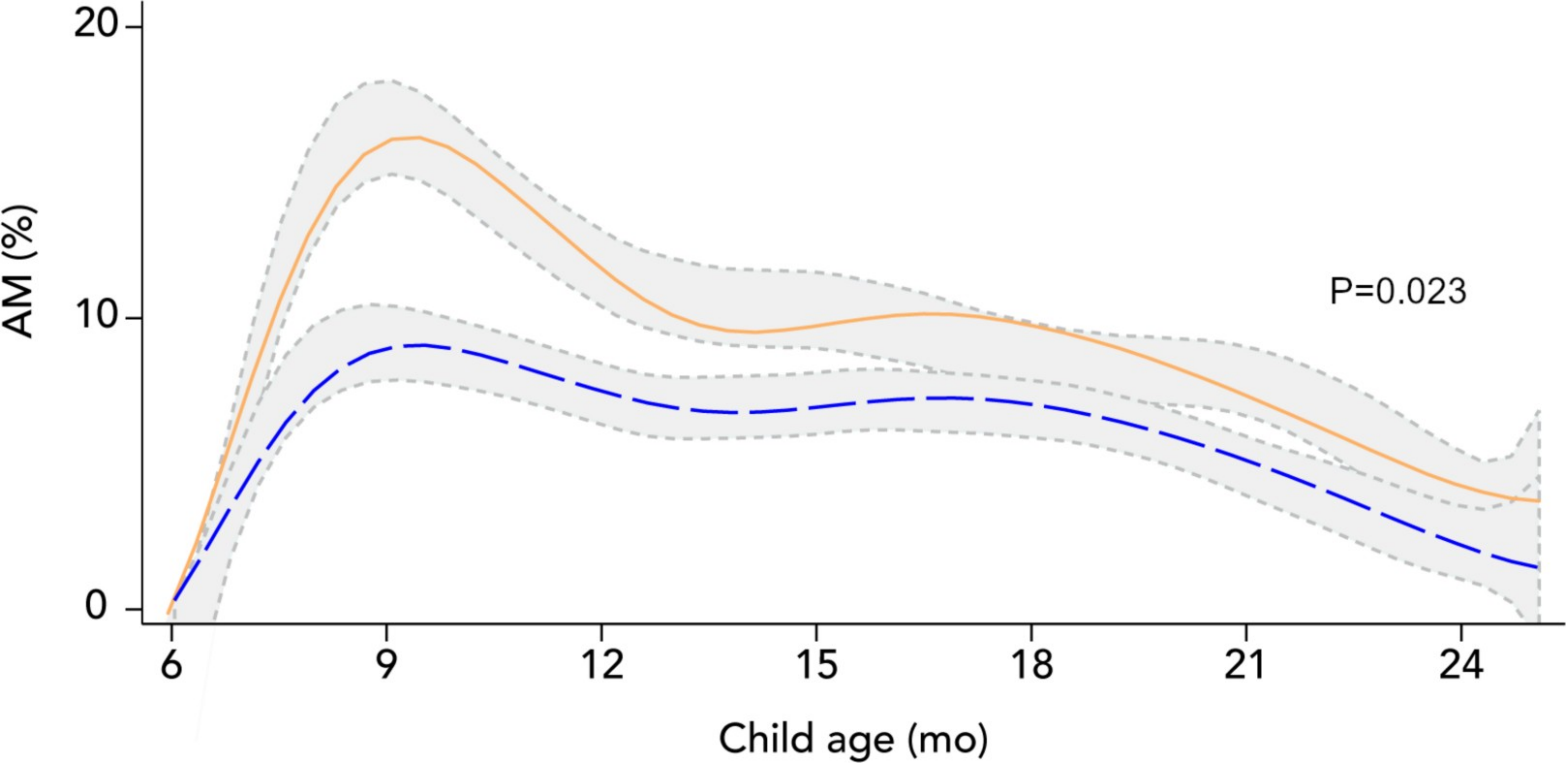
Effect modification of the intervention by child age on AM prevalence during follow-up of children enrolled in the longitudinal study (n = 10,282 child visits in the comparison arm and n = 10,236 in the intervention arm). The blue dashed line represents fitted values obtained from the regression model for the intervention arm. The orange solid line represents fitted values obtained from the same regression model but for the comparison arm. Gray areas represent 95% confidence bands of kernel-weighted local polynomial smoothed values by study arm using the observed data. Mixed-effects regression models with restricted cubic splines (knots at 9, 12, and 16 months of child age) were used with health center catchment area and child as random intercepts and health district, sampling strata, month of inclusion, child sex, whether the child was a first live birth or not, age splines, and intervention as fixed effects. A chunk Wald test was used to test the “age spline × intervention” interaction terms (*p*-value shown). AM, acute malnutrition.

The intervention led to a 29% (95% CI: 8%, 46%; *p* = 0.017) reduction in the incidence of a first AM episode (primary outcome) and reduced AM incidence by 31% (95% CI: 14%, 46%; *p* = 0.001) when considering all episodes (Table 7). Over the 18 months of follow-up, the intervention resulted in a 30% (95% CI: 12%, 44%; *p* = 0.002) lower longitudinal AM prevalence (secondary outcome). Similar, statistically significant reductions in incidence and longitudinal prevalence were found for MAM and SAM (S1 Table). No program impact on relapse rate was found. The Kaplan–Meier plot demonstrates that the difference between study arms in the probability of developing the first AM episode mainly occurred during the first 4 months of follow-up and then remained constant (Fig 4). The intervention modified the trend in AM prevalence over time, relative to the comparison arm (chunk test of interactions *p* = 0.023) (Fig 5). AM prevalence in the intervention arm was lower during the first months of follow-up relative to the comparison arm, but this initial difference disappeared as children grew older. The intervention also modified the change in WLZ with age (chunk test of interactions *p* < 0.001), leading to a significantly higher WLZ between 9 and 15 months of age relative to the comparison arm (S4 Fig). However, this impact gradually phased out after 15 months. The intervention had no impact on the changes in MUAC with age (S4 Fig).

**Table 7:** Effect of the intervention on the incidence, relapse, and longitudinal prevalence of AM assessed by longitudinal study.

	Comparison	Intervention	IRR/RR	95% CI	*p*-Value
**First episode of AM**					
N of children	567	565			
N of episodes/time at risk^a^, child-years	326/493	274/570			
Incidence (primary outcome)	0.66	0.48	0.71^b^	(0.54, 0.92)	0.017*
**All episodes of AM**					
N of children	567	565			
N of episodes/time at risk^c^, child-years	595/750	452/779			
Incidence	0.79	0.58	0.69^b^	(0.54, 0.86)	0.001
**Relapse episodes of AM**					
N of children	308	256			
N of episodes/time at risk^d^, child-years	269/257	178/209			
Relapse incidence	1.05	0.85	0.81^b^	(0.62, 1.1)	0.124
**Longitudinal prevalence AM**					
N of children	567	565			
Time AM/follow-up time, child-years	86/838	59/838			
Prevalence	10%	7.0%	0.70^e^	(0.56, 0.88)	0.002

**Abbreviations:** AM, acute malnutrition; CI, confidence interval; ICC, intracluster correlation coefficient; IRR, incidence rate ratio; RR, risk ratio.

*Statistically significant after correcting for multiple testing of primary outcomes, using a *p*_critical_ = 0.025 calculated using the Benjamini–Hochberg method. ICC for primary outcomes are presented in **S2 Table**.

^a^Time at risk included all consecutive days before the first episode of AM.

^b^IRR analyzed using a mixed-effects Poisson regression model with health center as random effect and sampling strata, health district, month of inclusion, child sex, child age, whether the child was the first liveborn, and intervention as fixed effects.

^c^Time at risk included all consecutive days before, between, and after episodes of AM.

^d^Time at risk included all consecutive days before, between, and after episodes of AM, starting after a first episode of AM.

^e^RR analyzed using a mixed-effects Poisson regression model with health center as random effect and sampling strata, health district, month of inclusion, child sex, whether the child was the first liveborn, and intervention as fixed effects.

## Discussion

Incorporating SQ-LNS distribution into monthly AM screenings and BCC sessions using a community-level platform had a large positive impact on AM screening coverage and reduced AM incidence in Mali. The intervention, however, had no impact on AM prevalence or treatment coverage.

Program impact was also found on several secondary outcomes, including attendance at monthly meetings with CHVs and coverage of BCC and SQ-LNS overall and through the monthly meetings. The intervention had a small impact on WLZ in the cross-sectional sample and age-dependent impacts on WLZ in the longitudinal sample. No impact was found on AM recovery, episode length, or relapse rate. An unintended negative impact on the proportion of AM episodes of children enrolled in the CMAM program (−10 pp in the intervention arm) was found.

### Impact on coverage of AM screening, BCC, and SQ-LNSs

The intervention led to a large positive impact on AM screening coverage (40 pp in the cross-sectional study and 28 pp in the longitudinal study), thus addressing one of the key barriers to CMAM program effectiveness. The observed monthly AM screening coverage at CHV meetings (63% overall; 42% through the meetings with CHVs at endline) in the intervention arm was high compared to other screening strategies. A much more intensive intervention (using door-to-door screening) in Mali, e.g., only achieved a 43% coverage of AM screening among 6- to 59-month–old children[15]. The companion study in Burkina Faso, which used a health facility platform, also found a positive impact on screening, but the overall AM screening coverage in the intervention arm was 48% at endline [16].

Larger attendance at monthly meetings with CHVs in the intervention compared to the comparison arm suggests that SQ-LNSs were a strong incentive for caregivers to attend the meetings. Although there is evidence of the incentive value of cash to promote health service utilization in the context of social safety net programs [38], no study to date has evaluated the impact of SQ-LNSs or any other food supplement as an incentive to participation in AM screening services. One study in neighboring Burkina Faso found that caregivers of children with MAM who were given food supplements had an approximately 13 pp higher attendance rate at weekly health center consultations compared to caregivers who received only patient-centered BCC. The latter group of caregivers consistently reported that they were not motivated to attend the consultations because the BCC included recommendations to prepare complementary foods with ingredients they could not afford to buy [39]. In India, adding small incentives (lentils and metal plates) to monthly child immunization camps led to significantly higher attendance rates (39%) to these camps compared to monthly immunization camps without these incentives (18%) [40].

Larger attendance at monthly screening meetings among the intervention arm also meant greater coverage of BCC compared to the comparison arm. The data show that caregivers almost always received the BCC when attending the monthly meetings, which shows that CHVs organized the meetings as per protocol for all attending caregivers. The BCC curriculum provided messages on IYCF practices and also addressed the topic of child AM and promoted the use of CMAM services, thereby potentially addressing two important barriers to CMAM treatment coverage: lack of awareness of child AM and of existing CMAM services [9]. Unfortunately, these efforts failed to translate into greater AM treatment coverage, as shown by our data.

Both the cross-sectional and longitudinal studies showed that many caregivers obtained SQ-LNSs outside the monthly CHV meeting **(Table 3)**. One-fifth of all CHVs reported having distributed the supplements to caregivers outside the monthly meeting. Nearly 60% of the caregivers who received SQ-LNSs outside monthly meetings reported having received the supplement at the CHV’s home and around 25% during a home visit by a CHV. These findings suggest that CHVs did not strictly enforce the conditionality of participating in the monthly meeting in order to receive SQ-LNSs. Nevertheless, the high SQ-LNS coverage shows that caregivers valued the SQ-LNSs as an important nutritional supplement for their child. Importantly, more than two-thirds of children (approximately 68%) who received SQ-LNSs outside the CHV meetings were screened for AM by the CHV, showing that most CHVs did not simply hand out the SQ-LNSs but also combined the distribution with screening. This finding also explains why overall screening coverage in the intervention arm was higher than screening coverage through monthly CHV meetings. The downside of this divergence from intervention protocol is that caregivers who received the SQ-LNSs outside the monthly meetings did not receive the BCC.

The longitudinal study shows that during the first months, the CHVs were not consistently organizing the village meetings. This lag explains why the coverage estimates for program services averaged over the 18-month follow-up were lower than those found in the cross-sectional endline survey. One notable exception was the SQ-LNS coverage, which was high from the onset. Through strengthened supervision of the CHVs in the villages by health center staff and the creation of nutrition support groups in all study villages, the coverage of all program services improved over time.

### Impact on AM treatment coverage

Contrary to our expectations, the positive impact of the intervention on AM screening coverage did not lead to higher AM treatment coverage. This finding was unexpected, especially given HKI’s extensive training and supervision of CHVs and efforts to support the AM treatment consultations through formative supervision. Clearly, there was a breakdown in the sequence of actions that need to take place to treat children with AM, i.e., screening and correct diagnosis; referral to the health center; confirmation of diagnosis at health center and enrollment in CMAM treatment; and treatment initiation, compliance, and completion. We used information available from our cross-sectional endline survey to identify some of the potential bottlenecks **(S5 Fig)**. Since the number of observations is small, these results are presented for exploratory purposes only.

First, since CHVs screened children based on MUAC and bilateral pitting edema only (as per protocol), they could have missed AM cases with an MUAC > 125 mm and a WLZ < −2. At the time of the endline survey, we found that nearly 29% of AM cases **(S5 Fig)** identified by the research team (who used the full set of screening criteria, including WLZ in addition to MUAC and edema) had an MUAC > 125 mm and a WLZ < 2 and could therefore not be detected by CHVs.

Second, less than half of the caregivers of AM children (as measured by the research team using MUAC and edema—the same criteria used by CHVs) who had been screened for AM in the month before the endline survey had been informed of their child’s AM **(S5 Fig)**. Given the time lag between the monthly CHV meeting and the endline measurement (between 1 to 30 days), it is possible that a proportion of these cases were not AM at the time of the monthly CHV meeting when AM screening occurred. It is unlikely, however, that this time lag explains the large discrepancy in diagnosis. Furthermore, it is also possible that CHVs missed cases because of MUAC measurement errors.

Third, caregivers in the intervention arm with an AM child not under treatment (AM assessed by the research team using MUAC and edema) were less likely to have been informed of their child’s AM status by the CHV than those in the comparison arm (**S5 Fig**). We believe that CHVs may have found it challenging to follow the protocol, which required referring the AM child to CMAM treatment and withholding SQ-LNSs. It is also possible that CHVs considered SQ-LNSs to be an acceptable AM treatment substitute. This hypothesis is supported by the finding that all caregivers of the very few cases (n = 4, **S5 Fig**) diagnosed with AM by CHVs in the intervention arm received SQ-LNSs. Direct observations of the monthly meetings documented several cases in which a CHV handed out SQ-LNSs to a mother of an AM child, explaining that if the child’s AM did not improve, he would refer her for treatment. Also, in the full sample of the intervention arm at endline, we found that 54% of the caregivers of children diagnosed by our research team as having AM reported having received SQ-LNSs in the month preceding the survey. The availability of SQ-LNSs might thus have created unintended interference with the AM screening to treatment pathway, which could explain why the intervention apparently reduced the percentage of AM children enrolled in the CMAM treatment program.

### Impact on child AM

The intervention was effective in lowering AM incidence (i.e., preventing the occurrence of new AM cases), and the magnitude of effect was large: the 29% lower risk of developing the first episode of AM found in the study is equivalent to preventing approximately 180 AM cases per 1,000 children every year. To our knowledge, there are no studies to date that have assessed the preventive impact of SQ-LNSs on the monthly incidence of AM. However, the observed impact seems plausible when compared to studies using fortified food supplements providing more calories (250–820 kcal per day) than the SQ-LNSs used in our study. In Niger, a 3-month supplementation study found that providing 92 g of LNS (approximately 500 kcal) daily reduced the incidence of wasting by 36% in children 6–60 months of age compared to a control group [41]. Also in Niger, a study providing a medium to large quantity of fortified flour or LNSs (250–500 kcal per day) to children aged 6–23 months in combination with a household cash transfer reduced MAM incidence by 40%–60% as compared to providing only the food supplement or only the cash [42].

In our study, the difference in AM incidence between study arms emerged between the age of 6 and 10 months, the peak of AM incidence in this population, and remained constant afterwards. A similar pattern was observed for AM prevalence assessed in the longitudinal study, with the largest difference between arms occurring between 6 and 10 months of age. Our findings suggest that this early preventive effect of the program can be attributed to the consumption of SQ-LNSs. The role of BCC was probably smaller, given the low coverage observed (only 10%–20%) between the age of 6 and 10 months. The impact of SQ-LNSs on preventing AM is biologically plausible: a daily dose of SQ-LNS provides 118 kcal, which represents 58% and 38% of the recommend energy intake from complementary foods for breastfed children 6–8 and 9–11 months old, respectively [20,43]. It is thus likely that the SQ-LNSs served as an energy supplement (while also providing significant amounts of essential micronutrients) and helped prevent AM at young ages. The lack of an effect at older ages might be because the energy contribution of the supplement at these ages became too small relative to requirements (representing only 22% of recommended energy from complementary foods for breastfed children between 12 and 23 months of age).

The intervention did not have a significant impact on AM prevalence at endline (cross-sectional study), but it did reduce the longitudinal AM prevalence and increase WLZ in the longitudinal study. The positive impact of 0.10 Z-scores on WLZ is similar to the effect of 0.07 Z-scores documented in a recent meta-analysis of interventions that offered complementary food supplements (including SQ-LNSs) with or without nutrition education in food insecure settings [44]. In the recent Water, Sanitation and Hygiene (WASH) benefits study in Bangladesh, the SQ-LNSs had an impact of 0.15 Z-scores on WLZ but did not have an effect on the prevalence of AM. No impact on these outcomes was found in the Kenya companion study [45,46].

We had hypothesized that the intervention would reduce AM prevalence through both the treatment and prevention pathways. Given the rather large impact of the intervention on AM screening coverage and incidence, we believe that the lack of a meaningful impact on AM prevalence is mainly due to the lack of impact on AM treatment coverage, as demonstrated in both study samples.

### Study strengths and limitations

This study used a rigorous randomized controlled evaluation design to study the impacts at scale of a program implemented through the Malian health system that targeted an estimated population of approximately 30,000 children 6–23 months of age in two health districts. The combined cross-sectional and longitudinal study designs allowed us to assess the program’s impact on AM prevalence and incidence. In addition, outcomes like participation in AM screening, BCC, and SQ-LNSs were assessed in two independently drawn samples. This strengthens the internal validity of the study findings. AM screening, BCC, SQ-LNSs, and treatment coverage, however, were based on caregiver recall, which might suffer from reporting bias. Since this bias is unlikely to be different between study arms, we do not believe it affected our impact estimates. The longitudinal study with monthly home visits offered detailed insights into the age dynamics of program participation, treatment coverage, and changes in child anthropometry. Given the closed cohort design that enrolled children over a 4-month window, however, the outcomes presented by child age also reflect seasonal variation. Again, because children in both study arms were enrolled during the same period, seasonal effects do not affect our impact estimates. For ethical reasons, our research teams referred AM cases identified at the monthly home surveys for treatment. As a result, the treatment coverage findings from the longitudinal study reflect screening and referral by both CHVs and research teams and are higher compared to those found in the cross-sectional surveys. Both study arms received the same number of monthly follow-up visits from our field teams, so there were no differences in screening and referral by field staff between study arms. However, this continuous follow-up might have reduced the caregivers‘ motivation to attend the monthly meetings with CHVs to have their children screened. The cross-sectional study was carried out in February–March, when AM prevalence tends to be lower than in August–September, which corresponds to the end of the rainy season when AM prevalence is the highest because of dwindling food stocks and the high rates of transmission of malaria [47]. Because of the lower AM prevalence during this season, the potential to find an impact was also lower. We did not conduct a process evaluation of CMAM treatment services organized at the health center level. Hence, we are limited in our assessment of possible bottlenecks or breakdowns in CMAM service delivery, quality, and uptake and their relationship with the observed AM treatment coverage results. Finally, an unexpected finding of the longitudinal study was that fewer AM episodes were enrolled for treatment in the intervention arm compared to the comparison arm. The AM episodes recorded by the longitudinal study, however, do not constitute a random population of episodes. Moreover, the intervention reduced the incidence of AM, so the episodes that did occur in the intervention arm may not be comparable (e.g., more difficult to prevent or associated with more severe morbidity) to those that occurred in the comparison arm. Therefore, we cannot exclude that the impact results on treatment coverage in the longitudinal study are influenced by the characteristics of the episodes used for this analysis.

### Using a community platform to integrate preventive services into AM screening

Comparing the findings from Mali with those from the companion study conducted in Burkina Faso [16] provides interesting insights on the strengths and limitations of different platforms to deliver integrated preventive and CMAM services. The Burkina Faso program used health-facility–based preventive well-baby consultations as the intervention platform (CNS or *Consultation du Nourrisson Sain* in French).

In both contexts, more than half of the intervention children attended the monthly meetings with CHVs in Mali or at in Burkina Faso, but the program’s impact on attendance was slightly larger in Mali (41 pp) compared to Burkina Faso (35 pp). This suggests that proximity to the CHV meetings, which were held at the community level in Mali, may have facilitated attendance compared to the CNS venue, which was at the health facility (usually farther away from caregivers’ home). Greater attendance at CHV meetings in Mali also led to a larger impact, compared to Burkina Faso, on SQ-LNSs (73 pp versus 46 pp) and AM screening (40 pp versus 25 pp) coverage at endline. This was in part due to greater attendance but also to the fact that in Mali, both SQ-LNSs and AM screening were also provided outside of the program’s platform. In Burkina Faso, the proportion of children with AM who received preventive SQ-LNSs (i.e., contrary to protocol) was markedly lower (26%) than in Mali (54%). The medical training of the health staff in charge of SQ-LNS distribution in Burkina Faso may have helped ensure fidelity of implementation of the SQ-LNS component of the intervention. The impact of PROMIS on BCC coverage was greater in Mali (38 pp) than in Burkina Faso (20 pp). In Burkina Faso, the centralized-facility–based platform attended to large numbers of caregivers and may have lacked the resources to provide all services, including regular and high-quality BCC for caregivers attending the CNS. Conversely, offering multiple program services like AM screening, BCC, and SQ-LNSs through a more decentralized community-based platform may allow more personalized services offered to a larger number of mothers but requires closer supervision and monitoring to ensure that implementation protocols are respected and quality of service delivery is achieved.

Notwithstanding the large improvements in screening coverage in our two study sites, 4 out of every 10 children failed to be screened for AM each month. The ideal frequency of AM screening remains a question because no official recommendation exists, but more frequent—e.g., monthly rather than quarterly—screenings are likely to ensure that a greater number of at-risk children are screened in a timely fashion. One potential approach to increase AM screening coverage that is receiving increasing attention is to transfer the responsibility to parents by providing them training and MUAC tapes. The approach could indeed increase screening coverage, especially if training was available to empower caregivers to acquire the necessary skills [48]. However, current evidence based on cross-sectional surveys and nonrandomized experiments suggests wide variations in caregiver involvement and thus coverage across settings [49]. The feasibility and effectiveness of this approach need to be evaluated using rigorous and comprehensive evaluation designs.

In both countries, the impact on AM screening coverage failed to translate into an impact on treatment coverage. These findings point to the presence of other caregiver-related barriers such as distance to treatment sites and caregiver opportunity costs related to attending treatment consultations [9] that were not addressed by this program. A recent study in Mali suggests that adding SAM treatment to the integrated community case management package delivered by CHWs at the community level is feasible and leads to better treatment adherence and similar treatment efficacy as health facility CMAM services [50,51].

### Conclusion

Integrating SQ-LNSs into community-level screening for AM and delivery of BCC in Mali improved AM screening coverage but did not have an impact on treatment coverage or the prevalence of AM. The intervention had a strong preventive impact on AM incidence and small impacts on mean WLZ and the longitudinal prevalence of AM. SQ-LNSs proved to be a powerful incentive for caregivers to participate in community-based services, and they appear to have contributed to the impact on child nutritional status.

Further research should test the operational feasibility and effectiveness of a community-based model that brings all elements of CMAM into a single community-level platform (including prevention, AM screening, CMAM referral, and treatment) in order to ease the burden of participation for caregivers. A key to the success of such an approach would be to establish a careful monitoring and supervision system to ensure high-quality implementation and service delivery without overburdening the system. Research should also assess the cost of implementing this type of community platform.

## Supporting information

S1 CONSORT checklist CONSORT Extension for Cluster Trials Checklist. CONSORT, Consolidated Standards of Reporting Trials.
(DOCX)

S1 Fig Caregiver attendance of monthly meetings with CHVs in the longitudinal study by child age and study arm. The blue dashed line represents fitted values from the regression model for the intervention arm (based on n = 9,434 child visits). Orange solid lines represents fitted values from the same regression model but for the comparison arm (based on n = 9,424 child visits). Gray areas represent 95% confidence bands of kernel-weighted local polynomial smoothed values by study arm using the observed data. Mixed-effects regression models with restricted cubic splines (knots at 9, 12, and 22 months of child age) were used with health center catchment area and child as random intercepts and health district, sampling strata, month of inclusion, child sex, whether the child was a first live birth or not, and age splines and intervention as fixed effects. A chunk Wald test was used to test the “age spline × intervention” interaction terms (*p*-values shown). CHV, community health volunteer.
(TIF)

S2 Fig **Total AM screening coverage (panel A) and AM screening coverage through the meeting with CHV (panel B) by child age and by study arm in the longitudinal study.** The blue dashed line represents fitted values obtained from the regression model for the intervention arm (based on n = 9,434 child visits). Orange solid lines represents fitted values obtained from the same regression model but for the comparison arm (based on n = 9,424 child visits). Gray areas represent 95% confidence bands of kernel-weighted local polynomial smoothed values by study arm using the observed data. Mixed-effects regression models with restricted cubic splines (knots at 9, 12, and 22 months of child age) were used with health center catchment area and child as random intercepts and health district, sampling strata, month of inclusion, child sex, whether the child was a first live birth or not, and age splines and intervention as fixed effects. A chunk Wald test was used to test the “age spline × intervention” interaction terms (p-values shown). AM, acute malnutrition; CHV, community health volunteer.
(TIF)

S3 Fig **Total BCC coverage (panel A) and BCC coverage through the meeting with CHVs (panel B) by child age and by study arm in the longitudinal study.** The blue dashed line represents fitted values obtained from the regression model for the intervention arm (n = 9,434 child visits). Orange solid lines represents fitted values obtained from the same regression model but for the comparison arm (n = 9,424 child visits). Gray areas represent 95% confidence bands of kernel-weighted local polynomial smoothed values by study arm using the observed data. Mixed-effects regression models with restricted cubic splines (knots at 9, 15, and 22 months of child age) were used with health center catchment area and child as random intercepts and health district, sampling strata, month of inclusion, child sex, whether the child was a first live birth or not, and age splines and intervention as fixed effects. A chunk Wald test was used to test the “age spline × intervention” interaction terms (*p*-values shown). BCC, behavior change communication; CHV, community health volunteer.
(TIF)

S4 Fig **Effect modification of the intervention by child age on monthly MUAC (panel A) and WLZ in children enrolled in the longitudinal study.** The blue dashed line represents fitted values obtained from the regression model for the intervention arm (n = 10,236 child visits). Orange solid lines represents fitted values obtained from the same regression model but for the comparison arm (n = 10,282 child visits). Gray areas represent 95% confidence bands of kernel-weighted local polynomial smoothed values by study arm using the observed data. Mixed-effects regression models with restricted cubic splines (knots at 9, 12, and 16 months of child age) were used with health center catchment area and child as random intercepts and health district, sampling strata, month of inclusion, child sex, whether the child was a first live birth or not, and age splines and intervention as fixed effects. A chunk Wald test was used to test the “age spline × intervention” interaction terms (*p*-values shown). MUAC, mid-upper arm circumference; WLZ, weight-for-length Z-score.
(TIF)

S5 Fig AM screening and diagnosis of AM cases (at the time of the endline survey) and SQ-LNS coverage at and outside of the monthly meeting with CHVs in the subsample of AM cases who did not receive any treatment product in the past month. ^a^Based on caregiver recall at the time of the survey. AM, acute malnutrition; CHV, community health volunteer; SQ-LNS, small-quantity lipid-based nutrient supplement.
(TIF)

S1 Table Effect of the intervention on the incidence, relapse, and longitudinal prevalence of moderate and severe AM assessed by longitudinal study. AM, acute malnutrition.
(DOCX)

S2 Table Intracluster correlation coefficients for primary outcomes. (DOCX)

S1 Text Study protocol for the PROMIS Mali study. PROMIS, Innovative Approaches for the Prevention of Childhood Malnutrition.
(PDF)

S2 Text Data analysis plan for PROMIS Mali. PROMIS, Innovative Approaches for the Prevention of Childhood Malnutrition.
(DOCX)

## Abbreviations

AM: acute malnutrition
BCC: behavior change communication
CHV: community health volunteer
CHW: community health worker
CI: confidence interval
CMAM: community-based management of acute malnutrition
CNS: Consultation du Nourrisson Sain
CONSORT: Consolidated Standards of Reporting Trials
FANTA: Food and Nutrition Technical Assistance III Project
FAO: Food and Agriculture Organization
FCS: fully conditional specification
FMPOS: Faculty of Medicine, Pharmacy and Onto-Stomatology
HKI: Helen Keller International
ICC: intracluster correlation coefficient
IYCF: infant and young child feeding
MAM: moderate acute malnutrition
MUAC: mid-upper arm circumference
pp: percentage point
PROMIS: Innovative Approaches for the Prevention of Childhood Malnutrition
RUSF: Ready-to-Use Supplementary Foods
RUTF: Ready-to-Use Therapeutic Foods
SAM: severe acute malnutrition
SQ-LNS: small-quantity lipid-based nutrient supplement
USAID: United States Agency for International Development
WASH: Water, Sanitation and Hygiene
WHO: World Health Organization
WLZ: weight-for-length Z-score

## References

[1] Unicef WHO, World Bank Group. Levels and Trends in Child Malnutrition In: Joint Child Malnutrition Estimates 2017 Geneva, Switzerland: WHO; 2017. p. 1–16. 10.1016/S0266-6138(96)90067-4

[2] Olofin I, McDonald CM, Ezzati M, Flaxman S, Black RE, Fawzi WW, et al Associations of suboptimal growth with all-cause and cause-specific mortality in children under five years: a pooled analysis of ten prospective studies. PLoS ONE. 2013;8: e64636 10.1371/journal.pone.0064636 23734210 PMC3667136

[3] Black RE, Victora CG, Walker SP, Bhutta ZA, Christian P, De Onis M, et al Maternal and child undernutrition and overweight in low-income and middle-income countries. Lancet. 2013;382: 427–451. 10.1016/S0140-6736(13)60937-X 23746772

[4] Collins S, Dent N, Binns P, Bahwere P, Sadler K, Hallam A. Management of severe acute malnutrition in children. Lancet. 2006;369: 1992–2000. 10.1016/S0140-6736(06)69443-9 17141707

[5] World Health Organization, World Food Programme, United Nations System Standing Committee on Nutrition, UNICEF. Community-based management of severe acute malnutrition Geneva: WHO; 2007.

[6] Briend A. Highly nutrient-dense spreads: a new approach to delivering multiple micronutrients to high-risk groups. Br J Nutr. 2001;85 Suppl 2: S175–9. 11509107

[7] Osendarp S, Rogers B, Ryan K, Manary M, Akomo P, Bahwere P, et al Ready-to-use foods for management of moderate acute malnutrition: Considerations for scaling up production and use in programs. Food Nutr Bull. 2015;36: S59–S64. 10.1177/15648265150361S110 25902616

[8] WHO. Technical note: Supplementary foods for the management of moderate acute malnutrition in infants and children 6–59 months of age Geneva: WHO; 2012 10.1227/00006123-199112000-00028

[9] Rogers E, Myatt M, Woodhead S, Guerrero S, Alvarez JL. Coverage of Community-Based Management of Severe Acute Malnutrition Programmes in Twenty-One Countries. PLoS ONE. 2015;10: 2012–2013. 10.1371/journal.pone.0128666 PMC4456359 26042827

[10] Guerrero S, Rogers E. Is community-based treatment of severe acute malnutrition (SAM) at scale capable of meeting global needs? London: Coverage Monitoring Network; 2013 10.1179/146531207225022644

[11] Bliss JR, Njenga M, Stoltzfus RJ, Pelletier DL. Stigma as a barrier to treatment for child acute malnutrition in Marsabit County, Kenya. Matern Child Nutr. 2016;12: 125–138. 10.1111/mcn.12198 25989353 PMC6860141

[12] Guerrero S, Myatt M, Collins S. Determinants of coverage in Community-based Therapeutic Care programmes: Towards a joint quantitative and qualitative analysis. Disasters. 2010;34: 571–585. 10.1111/j.1467-7717.2009.01144.x 20002705

[13] Puett C, Guerrero S. Barriers to access for severe acute malnutrition treatment services in Pakistan and Ethiopia: A comparative qualitative analysis. Public Health Nutr. 2015;18: 1873–1882. 10.1017/S1368980014002444 26017477 PMC10271649

[14] Bernabé BP. Community based Management of Acute Malnutrition in Koutiala district: a coverage assessment Bamako: Ministry of Public Health and Hygiene; 2013 Available from: http://www.coverage-monitoring.org/wp-content/uploads/2014/01/Rapport-SQUEAC_Koutiala-VF.pdf

[15] Nyirandutiye DH, Ag Iknane A, Fofana A, Brown KH. Screening for Acute Childhood Malnutrition during the National Nutrition Week in Mali Increases Treatment Referrals. Mock N, editor. PLoS ONE. 2011;6: e14818 10.1371/journal.pone.0014818 21731602 PMC3121698

[16] Becquey E, Huybregts L, Zongrone A, Le Port A, Leroy JL, Rawat R, et al Impact on child acute malnutrition of integrating a preventive nutrition package into facility-based screening for acute malnutrition during well-baby consultation: A cluster-randomized controlled trial in Burkina Faso. PLoS Med. 2019;16(8): e1002877 10.1371/journal.pmed.1002877 PMC6711504 31454347

[17] Huybregts L, Becquey E, Zongrone A, Le Port A, Khassanova R, Coulibaly L, et al The impact of integrated prevention and treatment on child malnutrition and health: the PROMIS project, a randomized control trial in Burkina Faso and Mali. BMC Public Health. 2017;17: 237 10.1186/s12889-017-4146-6 28274214 PMC5343313

[18] Institut National de la Statistique. Enquête Nutritionnelle et de Mortalité Rétrospective suivant la méthodologie SMART, Mali, 2018. Bamako: Institut National de la Statistique; 2018 Available from: https://fscluster.org/mali/document/cluster-nut-enquete-nationale.

[19] Guyon A, Quinn V, Nielsen J, Stone-Jimenez M. Essential Nutrition Actions and Essential Hygiene Actions Training Guide: Community Workers. Washington, DC: Core Group; 2015.

[20] Arimond M, Zeilani M, Jungjohann S, Brown KH, Ashorn P, Allen LH, et al Considerations in developing lipid-based nutrient supplements for prevention of undernutrition: Experience from the International Lipid-Based Nutrient Supplements (iLiNS) Project. Matern Child Nutr. 2015;11: 31–61. 10.1111/mcn.12049 23647784 PMC6860325

[21] Guyon A, Quinn V, Nielsen J, Stone-Jimenez M. Essential Nutrition Actions and Essential Hygiene Actions Training Guide: Health Workers and Nutrition Managers. Washington, DC: Core Group; 2015.

[22] Macro ORC. Enquête Démographique et de Santé du Mali EDSM-V Rapport Préliminaire. Calverton, MD: ICF International; 2013.

[23] Leroy JL. ZSCORE06: Stata command for the calculation of anthropometric z-scores using the 2006 WHO child growth standards [computer program]. 2011. Available from: http://econpapers.repec.org/software/bocbocode/s457279.htm. [cited 2019 July 21]

[24] WHO Multicentre Growth Reference Study Group. WHO child growth standards: Length/height-for-age, weight-for-age, weight-for-length, weight-for-height and body mass index-for-age: methods and development Geneva: WHO; 2006.

[25] Cogill B. Anthropometric indicators measurement guide Washington, DC: FANTA; 2003.

[26] Vyas S, Kumaranayake L. Constructing socio-economic status indices: How to use principal components analysis. Health Policy Plan. 2006;21: 459–468. 10.1093/heapol/czl029 17030551

[27] Benjamini Y, Hochberg Y. Controlling the False Discovery Rate: A Practical and Powerful Approach to Multiple Testing. J R Stat Soc Ser B. 1995;57: 289–300.

[28] Harrell F. E. J. Regression Modeling Strategies: With Applications to Linear Models, Logistic Regression, and Survival Analysis. New York: Springer; 2001.

[29] Nurminen M. To use or not to use the odds ratio in epidemiologic analyses? Eur J Epidemiol. 1995;11: 365–371. 10.1007/BF01721219 8549701

[30] Zou G. A Modified Poisson Regression Approach to Prospective Studies with Binary Data. Am J Epidemiol. 2004;159: 702–706. 10.1093/aje/kwh090 15033648

[31] Cummings P. The Relative Merits of Risk Ratios and Odds Ratios. Arch Pediatr Adolesc Med. 2009;163: 438–445. 10.1001/archpediatrics.2009.31 19414690

[32] Nevalainen J, Kenward MG, Virtanen SM. Missing values in longitudinal dietary data:Amultiple imputation approach based on a fully conditional specification. Stat Med. 2009;28: 3657–3669. 10.1002/sim.3731 19757484

[33] Welch CA, Petersen I, Bartlett JW, White IR, Marston L, Morris RW, et al Evaluation of two-fold fully conditional specification multiple imputation for longitudinal electronic health record data. Stat Med. 2014;33: 3725–3737. 10.1002/sim.6184 24782349 PMC4285297

[34] van Buuren S, Boshuizen HC, Knook DL. Multiple imputation of missing blood pressure covariates in survival analysis. Stat Med. 1999;18: 681–694. 10.1002/(SICI)1097-0258(19990330)18:6<681::AID-SIM71>3.0.CO;2-R 10204197

[35] Coates J, Swindale A, Bilinsky P. Household Food Insecurity Access Scale (HFIAS) for measurement of food access: indicator guide Washington, DC: FANTA; 2007 10.1007/s13398-014-0173-7.2

[36] Martin-Prevel Y, Allemand P, Wiesmann D, Arimond M, Ballard T, Deitchler M, et al Moving forward on choosing a standard operational indicator of women’s dietary diversity Rome: FAO; 2015.

[37] WHO. Indicators for assessing infant and young child feeding practices Geneva: WHO; 2008. ISBN 978 92 4 159975 7

[38] Bastagli F, Hagen-zanker J, Harman L, Barca V, Sturge G, Schmidt T, et al Cash transfers: what does the evidence say? A rigorous review of programme impact and of the role of design and implementation features London: Overseas Development Institute; 2016.

[39] Nikiema L, Huybregts L, Kolsteren P, Lanou H, Tiendrebeogo S, Bouckaert K, et al Treating moderate acute malnutrition in first-line health services: an effectiveness cluster-randomized trial in Burkina Faso. Am J Clin Nutr. 2014;100: 241–249. 10.3945/ajcn.113.072538 24808482

[40] Banerjee AV, Duflo E, Glennerster R, Kothari D. Improving immunisation coverage in rural India: clustered randomised controlled evaluation of immunisation campaigns with and without incentives. BMJ. 2010;340: c2220 10.1136/bmj.c2220 20478960 PMC2871989

[41] Isanaka S, Nombela N, Djibo A, Poupard M, Van Beckhoven D, Gaboulaud V, et al Effect of preventive supplementation with ready-to-use therapeutic food on the nutritional status, mortality, and morbidity of children aged 6 to 60 months in Niger: A cluster randomized trial. JAMA—J Am Med Assoc. 2009;301: 277–285. 10.1001/jama.2008.1018 19155454 PMC3144630

[42] Langendorf C, Roederer T, de Pee S, Brown D, Doyon S, Mamaty AA, et al Preventing Acute Malnutrition among Young Children in Crises: A Prospective Intervention Study in Niger. PLoS Med. 2014;11 10.1371/journal.pmed.1001714 25180584 PMC4152259

[43] Dewey KG, Brown KH. Update on technical issues concerning complementary feeding of young children in developing countries and implications for intervention programs. Food Nutr Bull. 2003;24: 5–28. 10.1177/156482650302400102 12664525

[44] Panjwani A, Heidkamp R. Complementary Feeding Interventions Have a Small but Significant Impact on Linear and Ponderal Growth of Children in Low- and Middle-Income Countries: A Systematic Review and Meta-Analysis. J Nutr. 2017;147: 2169S–2178S. 10.3945/jn.116.243857 28904113

[45] Null C, Stewart CP, Pickering AJ, Dentz HN, Arnold BF, Arnold CD, et al Effects of water quality, sanitation, handwashing, and nutritional interventions on diarrhoea and child growth in rural Kenya: a cluster-randomised controlled trial. Lancet Glob Heal. 2018;6(3):e316–e329. 10.1016/S2214-109X(18)30005-6 29396219 PMC5809717

[46] Luby SP, Rahman M, Arnold BF, Unicomb L, Ashraf S, Winch PJ, et al Effects of water quality, sanitation, handwashing, and nutritional interventions on diarrhoea and child growth in rural Bangladesh: A cluster randomised controlled trial. Lancet Glob Heal. 2018;6(3):e302–315. 10.1016/S2214-109X(17)30490-4 29396217 PMC5809718

[47] Page A-L, de Rekeneire N, Sayadi S, Aberrane S, Janssens A-C, Rieux C, et al Infections in Children Admitted with Complicated Severe Acute Malnutrition in Niger. PLoS ONE. 2013;8: e68699 10.1371/journal.pone.0068699 23874731 PMC3714292

[48] Blackwell N, Myatt M, Allafort-Duverger T, Balogoun A, Ibrahim A, Briend A. Mothers Understand And Can do it (MUAC): a comparison of mothers and community health workers determining mid-upper arm circumference in 103 children aged from 6 months to 5 years. Arch Public Heal. 2015;73: 26 10.1186/s13690-015-0074-z 25992287 PMC4436117

[49] Bliss J, Lelijveld N, Briend A, Kerac M, Manary M, McGrath M, et al Use of Mid-Upper Arm Circumference by Novel Community Platforms to Detect, Diagnose, and Treat Severe Acute Malnutrition in Children: A Systematic Review. Glob Heal Sci Pract. 2018;6: 552–564. 10.9745/GHSP-D-18-00105 30185435 PMC6172115

[50] Alvarez Morán JL, Alé FGB, Rogers E, Guerrero S. Quality of care for treatment of uncomplicated severe acute malnutrition delivered by community health workers in a rural area of Mali. Matern Child Nutr. 2018;14: 1–7. 10.1111/mcn.12449 28378463 PMC6866144

[51] Alvarez Morán JL, Alé GBF, Charle P, Sessions N, Doumbia S, Guerrero S. The effectiveness of treatment for Severe Acute Malnutrition (SAM) delivered by community health workers compared to a traditional facility based model. BMC Health Serv Res. 2018;18: 207 10.1186/s12913-018-2987-z 29580238 PMC5870488

