# Supplementary figures and images for: Impact on child acute malnutrition of integrating small-quantity lipid-based nutrient supplements into community-level screening for acute malnutrition: A cluster-randomized controlled trial in Mali
Source: PLoS Med. 2019 Aug 27;16(8):e1002892. doi: 10.1371/journal.pmed.1002892 (PMC6711497; doi:10.1371/journal.pmed.1002892)

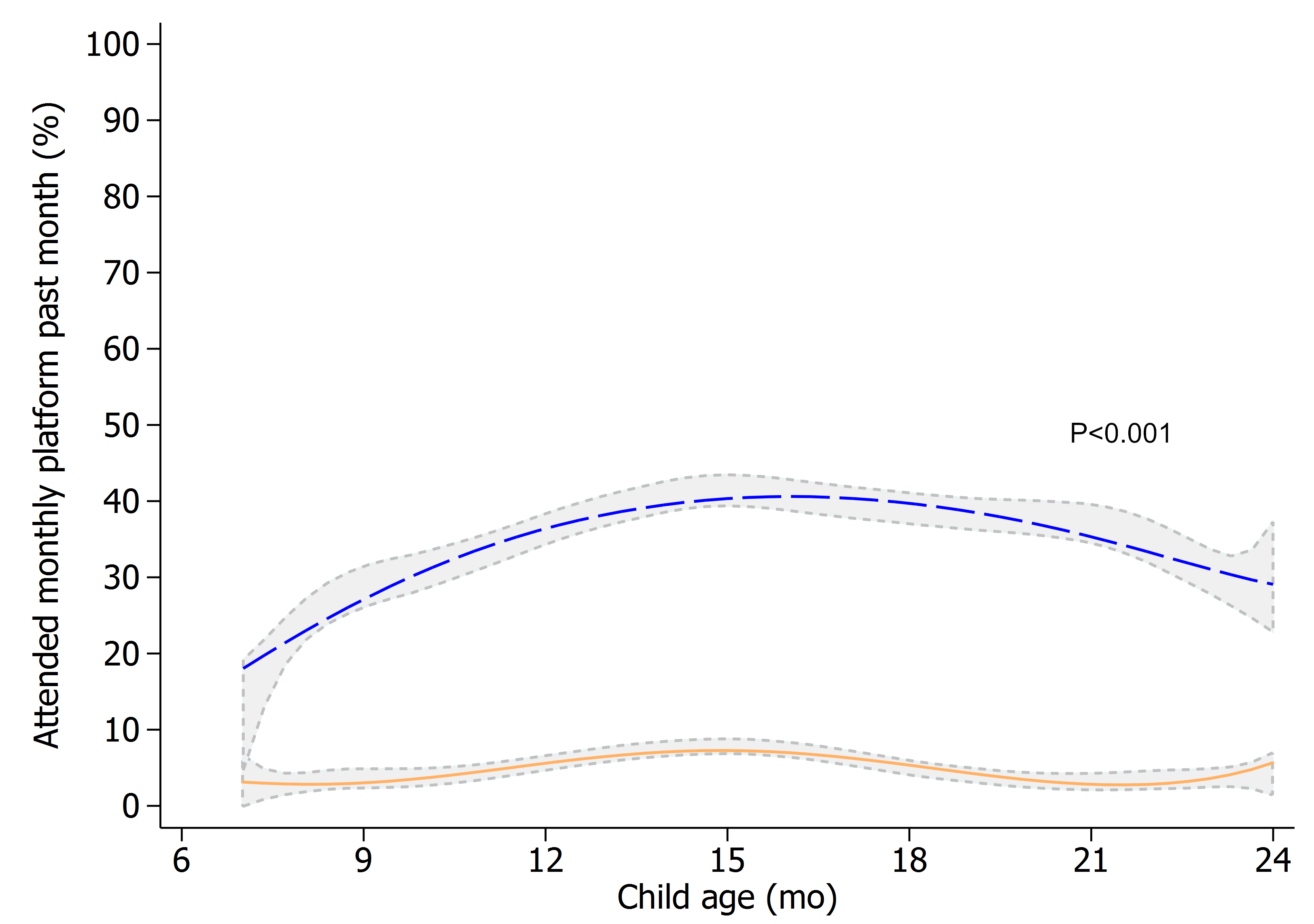

Supplement: S1 Fig — The blue dashed line represents fitted values from the regression model for the intervention arm (based on n = 9,434 child visits). Orange solid lines represents fitted values from the same regression model but for the comparison arm (based on n = 9,424 child visits). Gray areas represent 95% confidence bands of kernel-weighted local polynomial smoothed values by study arm using the observed data. Mixed-effects regression models with restricted cubic splines (knots at 9, 12, and 22 months of child age) were used with health center catchment area and child as random intercepts and health district, sampling strata, month of inclusion, child sex, whether the child was a first live birth or not, and age splines and intervention as fixed effects. A chunk Wald test was used to test the “age spline × intervention” interaction terms (p-values shown). CHV, community health volunteer. (TIF) [file pmed.1002892.s002.tif]

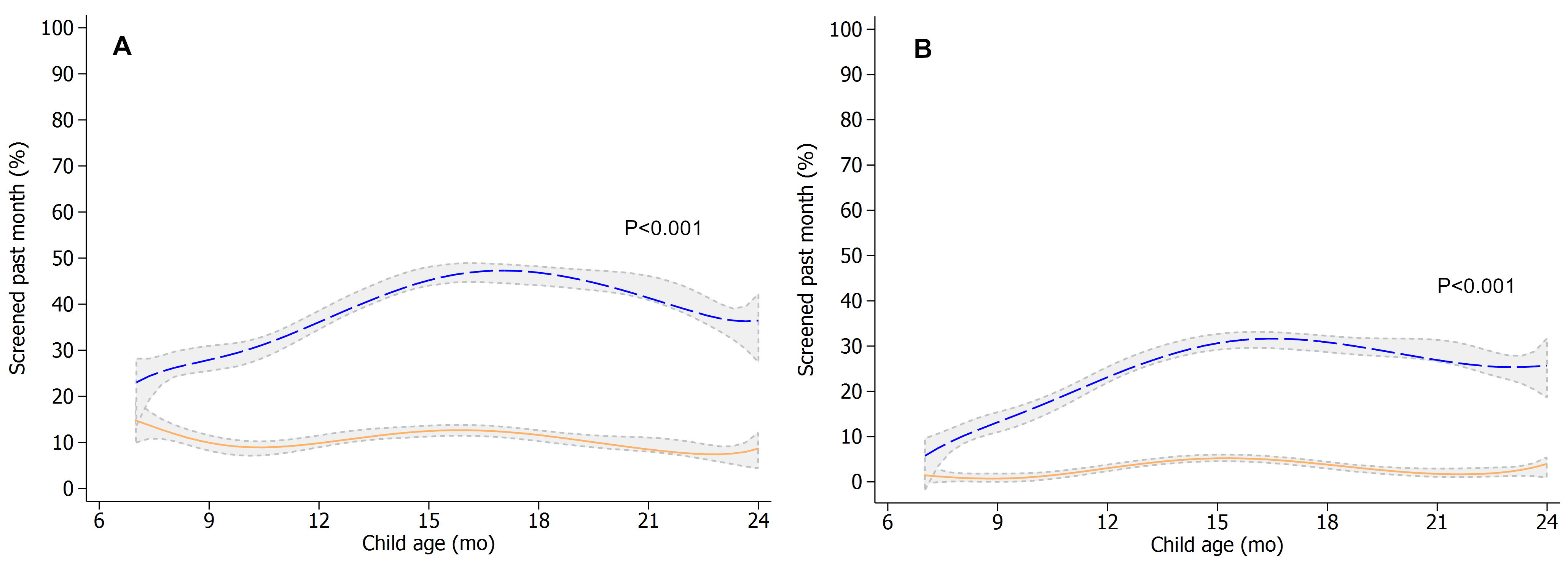

Supplement: S2 Fig — Total AM screening coverage (panel A) and AM screening coverage through the meeting with CHV (panel B) by child age and by study arm in the longitudinal study. The blue dashed line represents fitted values obtained from the regression model for the intervention arm (based on n = 9,434 child visits). Orange solid lines represents fitted values obtained from the same regression model but for the comparison arm (based on n = 9,424 child visits). Gray areas represent 95% confidence bands of kernel-weighted local polynomial smoothed values by study arm using the observed data. Mixed-effects regression models with restricted cubic splines (knots at 9, 12, and 22 months of child age) were used with health center catchment area and child as random intercepts and health district, sampling strata, month of inclusion, child sex, whether the child was a first live birth or not, and age splines and intervention as fixed effects. A chunk Wald test was used to test the “age spline × intervention” interaction terms (p-values shown). AM, acute malnutrition; CHV, community health volunteer. (TIF) [file pmed.1002892.s003.tif]

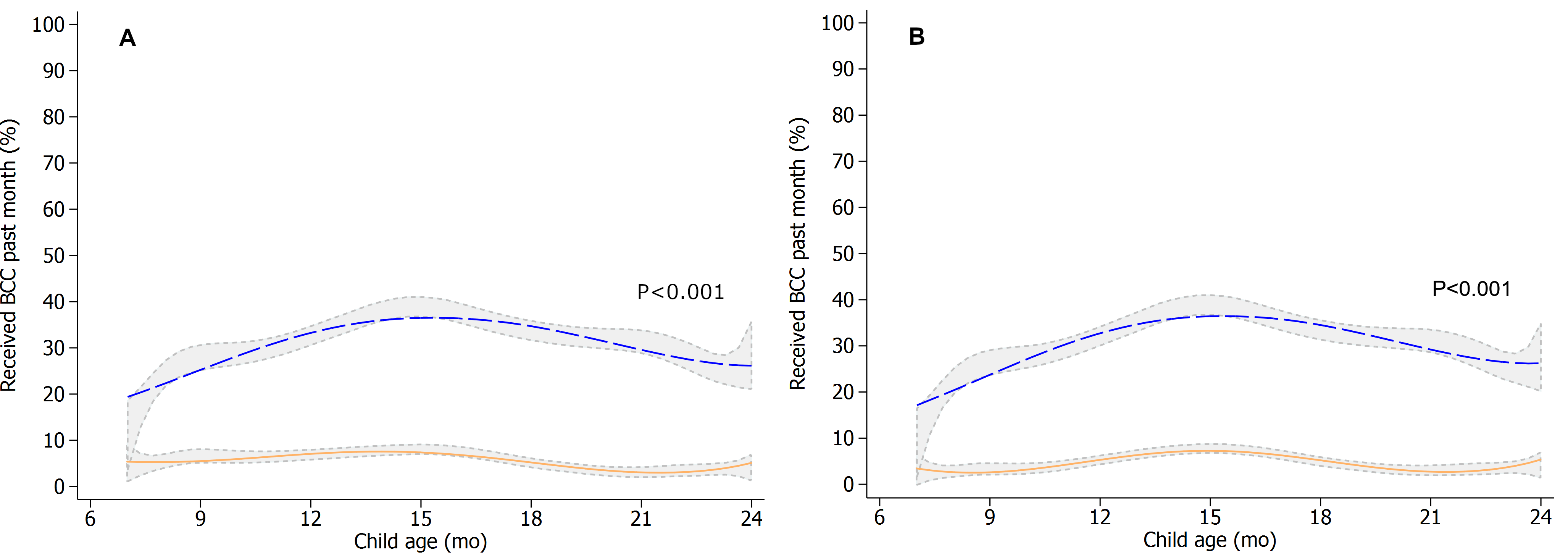

Supplement: S3 Fig — Total BCC coverage (panel A) and BCC coverage through the meeting with CHVs (panel B) by child age and by study arm in the longitudinal study. The blue dashed line represents fitted values obtained from the regression model for the intervention arm (n = 9,434 child visits). Orange solid lines represents fitted values obtained from the same regression model but for the comparison arm (n = 9,424 child visits). Gray areas represent 95% confidence bands of kernel-weighted local polynomial smoothed values by study arm using the observed data. Mixed-effects regression models with restricted cubic splines (knots at 9, 15, and 22 months of child age) were used with health center catchment area and child as random intercepts and health district, sampling strata, month of inclusion, child sex, whether the child was a first live birth or not, and age splines and intervention as fixed effects. A chunk Wald test was used to test the “age spline × intervention” interaction terms (p-values shown). BCC, behavior change communication; CHV, community health volunteer. (TIF) [file pmed.1002892.s004.tif]

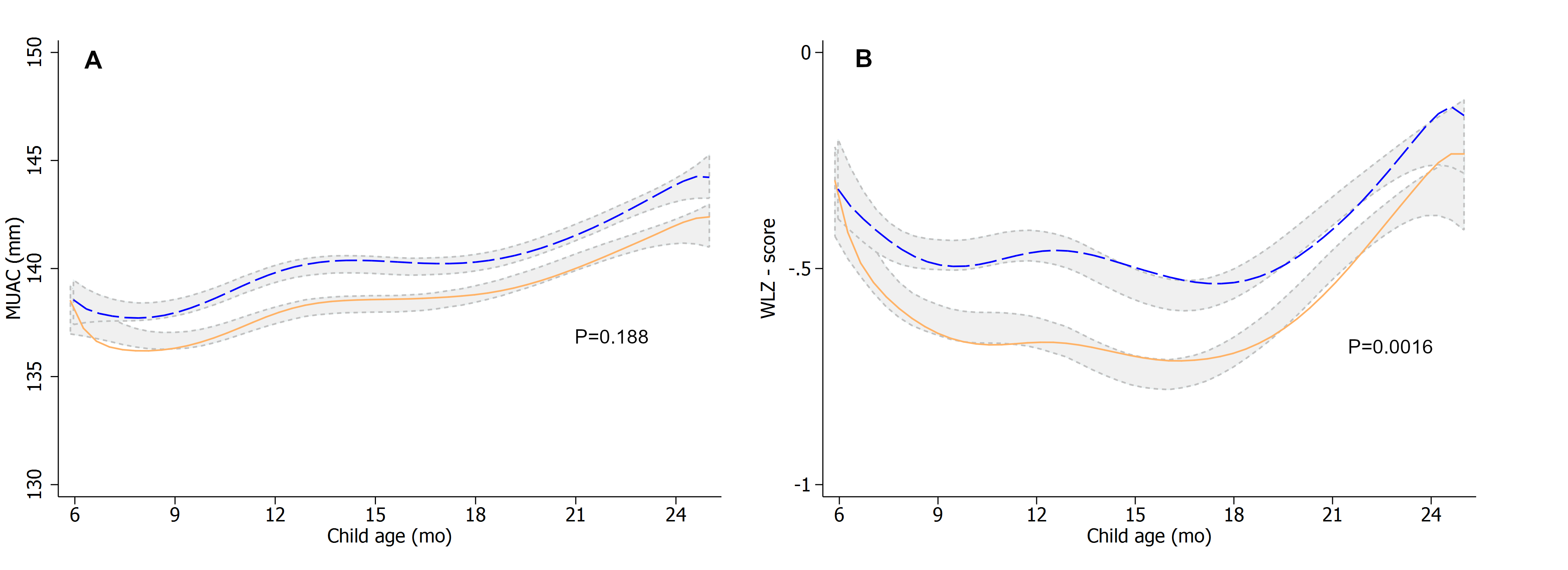

Supplement: S4 Fig — Effect modification of the intervention by child age on monthly MUAC (panel A) and WLZ in children enrolled in the longitudinal study. The blue dashed line represents fitted values obtained from the regression model for the intervention arm (n = 10,236 child visits). Orange solid lines represents fitted values obtained from the same regression model but for the comparison arm (n = 10,282 child visits). Gray areas represent 95% confidence bands of kernel-weighted local polynomial smoothed values by study arm using the observed data. Mixed-effects regression models with restricted cubic splines (knots at 9, 12, and 16 months of child age) were used with health center catchment area and child as random intercepts and health district, sampling strata, month of inclusion, child sex, whether the child was a first live birth or not, and age splines and intervention as fixed effects. A chunk Wald test was used to test the “age spline × intervention” interaction terms (p-values shown). MUAC, mid-upper arm circumference; WLZ, weight-for-length Z-score. (TIF) [file pmed.1002892.s005.tif]

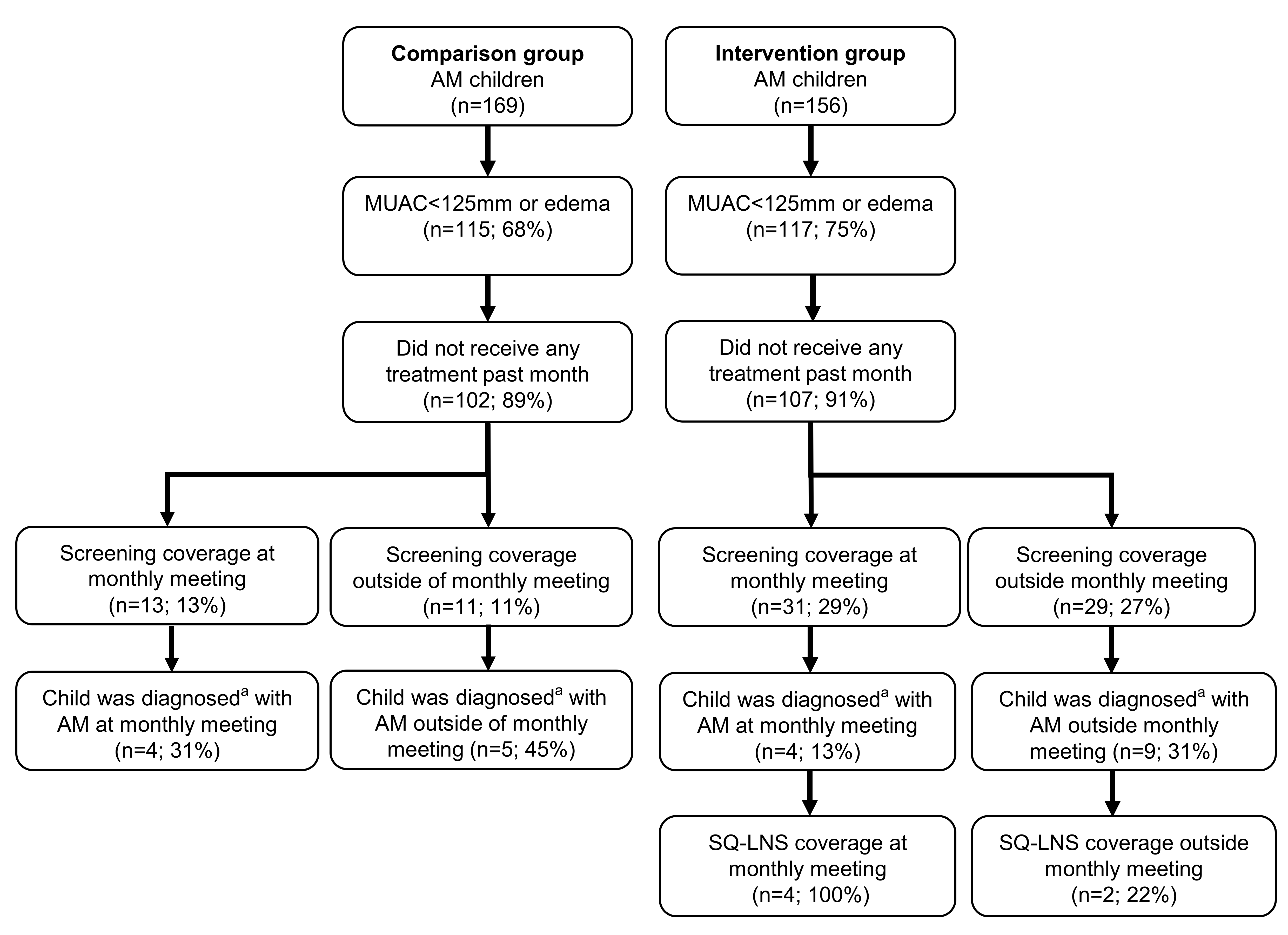

Supplement: S5 Fig — aBased on caregiver recall at the time of the survey. AM, acute malnutrition; CHV, community health volunteer; SQ-LNS, small-quantity lipid-based nutrient supplement. (TIF) [file pmed.1002892.s006.tif]
