# Supplementary material for: Impact on child acute malnutrition of integrating small-quantity lipid-based nutrient supplements into community-level screening for acute malnutrition: A cluster-randomized controlled trial in Mali
Source: PLoS Med. 2019 Aug 27;16(8):e1002892. doi: 10.1371/journal.pmed.1002892 (PMC6711497; doi:10.1371/journal.pmed.1002892)
Supplement: S1 Table — AM, acute malnutrition. (DOCX) [file pmed.1002892.s007.docx]

**S1 Table: Effect of the intervention on the incidence, relapse and longitudinal prevalence of moderate and severe acute malnutrition assessed by longitudinal study**

|  | Comparison | Intervention | IRR/RR | 95% CI | *p*-value |
| --- | --- | --- | --- | --- | --- |
| **Moderate Acute Malnutrition^a^** |  |  |  |  |  |
| **First episode of MAM** |  |  |  |  |  |
| N of children | 567 | 565 |  |  |  |
| N of episodes / time at risk^b^ , child-years | 289/535 | 243/604 |  |  |  |
| Incidence | 0.54 | 0.40 | 0.67 ^c^ | (0.53, 0.84) | 0.001 |
|  |  |  |  |  |  |
| **All episodes of MAM** |  |  |  |  |  |
| N of children | 567 | 565 |  |  |  |
| N of episodes / time at risk^d^ , child-years | 508/771 | 389/791 |  |  |  |
| Incidence | 0.66 | 0.49 | 0.70 ^c^ | (0.54 ,0.61) | 0.005 |
|  |  |  |  |  |  |
| **Relapse episodes of MAM** |  |  |  |  |  |
| N of children | 274 | 229 |  |  |  |
| N of episodes / time at risk^e^, child-years | 219/236 | 146/187 |  |  |  |
| Relapse incidence | 0.93 | 0.78 | 0.85 ^c^ | (0.65, 1.12) | 0.25 |
|  |  |  |  |  |  |
| **Longitudinal prevalence MAM** |  |  |  |  |  |
| N of children | 576 | 565 |  |  |  |
| Time being AM / Follow-up time, child-years | 64/838 | 47/838 |  |  |  |
| Prevalence | 7.62% | 5.57% | 0.75 ^f^ | (0.61, 0.91) | 0.005 |
|  |  |  |  |  |  |
| **Severe Acute Malnutrition** |  |  |  |  |  |
| **First episode of SAM** |  |  |  |  |  |
| N of children | 567 | 565 |  |  |  |
| N of episodes / time at risk^b^ , child-years | 104/741 | 72/777 |  |  |  |
| Incidence | 0.14 | 0.09 | 0.65 ^c^ | (0.44, 0.96) | 0.031 |
|  |  |  |  |  |  |
| **All episodes of SAM** |  |  |  |  |  |
| N of children | 567 | 565 |  |  |  |
| N of episodes / time at risk^d^ , child-years | 120/816 | 78/827 |  |  |  |
| Incidence | 0.15 | 0.09 | 0.63 ^c^ | (0.44, 0.90) | 0.011 |
|  |  |  |  |  |  |
| **Relapse episodes of SAM** |  |  |  |  |  |
| N of children | 93 | 66 |  |  |  |
| N of episodes / time at risk^e^, child-years | 16/75 | 6/50 |  |  |  |
| Relapse incidence | 0.21 | 0.12 | 0.56 ^c^ | (0.17, 1.75) | 0.316 |
|  |  |  |  |  |  |
| **Longitudinal prevalence SAM** |  |  |  |  |  |
| N of children | 576 | 565 |  |  |  |
| Time being AM / Follow-up time, child-years | 21/838 | 12/838 |  |  |  |
| Prevalence | 2.53% | 1.39% | 0.57 ^f^ | (0.36, 0.90) | 0.017 |

CI, confidence interval; IRR, incidence rate ratio; MAM, moderate acute malnutrition; RR, risk ratio; SAM, severe acute malnutrition

^a^ Children who suffered from SAM and qualified as MAM during the recovery process were not included in the MAM incidence estimation

^b^ time at risk included all consecutive days before the first episode of AM

^c^ Incidence rate ratio (IRR) analyzed using a mixed-effects Poisson regression model with health center as random effect and sampling strata, health district, month of inclusion, child sex, child age, whether the child was the first liveborn and intervention as fixed effects

^d^ time at risk included all consecutive days before, between and after episodes of AM

^e^ time at risk included all consecutive days before, between and after episodes of AM, starting after a first episode of AM

^f^ Risk ratio (RR) analyzed using a mixed-effects Poisson regression model with health center as random effect and sampling stratum, health district, month of inclusion, child sex, whether the child was the first liveborn and intervention as fixed effects.
