# Supplementary material for: Impact on child acute malnutrition of integrating small-quantity lipid-based nutrient supplements into community-level screening for acute malnutrition: A cluster-randomized controlled trial in Mali
Source: PLoS Med. 2019 Aug 27;16(8):e1002892. doi: 10.1371/journal.pmed.1002892 (PMC6711497; doi:10.1371/journal.pmed.1002892)
Supplement: S2 Table — (DOCX) [file pmed.1002892.s008.docx]

**S2 Table: Intra-cluster correlation coefficients for primary study outcomes**

|  |  | Total | Comparison | Intervention |
| --- | --- | --- | --- | --- |
| Endline survey | |  |  |  |
|  | AM screening coverage | 0.264 | 0.09 | 0.134 |
|  | AM treatment coverage | 0.005 | <0.001 | 0.016 |
|  | AM prevalence | <0.001 | <0.001 | <0.001 |
|  |  |  |  |  |
| Longitudinal study | |  |  |  |
|  | AM screening coverage | 0.171 | 0.024 | 0.09 |
|  | AM treatment coverage | <0.001 | <0.001 | 0.018 |
|  | AM Incidence | 0.022 | 0.015 | 0.009 |
