# Supplementary material for: Impact on child acute malnutrition of integrating small-quantity lipid-based nutrient supplements into community-level screening for acute malnutrition: A cluster-randomized controlled trial in Mali
Source: PLoS Med. 2019 Aug 27;16(8):e1002892. doi: 10.1371/journal.pmed.1002892 (PMC6711497; doi:10.1371/journal.pmed.1002892)
Supplement: S1 Text — PROMIS, Innovative Approaches for the Prevention of Childhood Malnutrition. (PDF) [file pmed.1002892.s009.pdf]

The PROMIS study

The effect of integrated prevention and treatment on child malnutrition and health in Mali: a cluster randomized intervention study

IFPRI & Helen Keller International

THE PROMIS PROJECT

SUMMARY SHEET

**Country:**	Mali
**Type of project:**	Research project<br>Cluster-randomized, non-masked, community-based, effectiveness trial
**Project Title:**	**The effect of integrated prevention and treatment on child malnutrition in Mali**
**Version:**	Version 1.2, dated September 20,2014
**Project codes:**	PROMIS-Mali<br>ClinTrials.Gov: registration to be completed once IRB clearance is obtained
**Project summary:**	The overall objective of this project is to lower the prevalence of acute and chronic malnutrition through an integration of preventive BCC/SQ-LNS and enhanced contact coverage and treatment referral and adherence in the Bla and San health districts, Ségou region, Mali, West-Africa.<br>The evaluation will be based on a cluster randomized controlled trial randomizing 48 health center catchment areas and will adopt 2 different evaluation designs: i) a comparison of the control (n=1,152) and intervention (n=1,152) groups through a longitudinal follow-up of 18 months intervention; ii) an endline comparison between intervention (n=552) and control study groups (n=552) on primary and secondary outcomes.<br>The main impact outcomes are:<br>- Prevalence of child acute malnutrition (wasting) at endline;<br>- Incidence of child acute malnutrition over 18 months of intervention;<br>- Coverage of screening for acute malnutrition<br>- Coverage of community-based case-finding and treatment of acute malnutrition over 18 months of intervention and at endline.
**Project Duration:**	27 months
**Starting Date:**	December 2014
**Completion Date:**	February 2017

**Executing Agency:**	Helen Keller International Mali
**Sponsor :**	International Food Policy Research Institute
**Project Contacts:**	Marie Ruel – IFPRI USA – m.ruel@cgiar.org<br>Rahul Rawat – IFPRI Dakar – r.rawat@cgiar.org<br>Lazare Coulibaly – HKI Mali – lcoulibaly@hki.org

STATEMENT OF COMPLIANCE & CONFIDENTIALITY

By signing this protocol, the Principal Investigator and the Sponsor acknowledge and agree:

This protocol contains all necessary information for conducting this study. The Principal Investigator will conduct this study as detailed herein and will make every reasonable effort to complete the study within the time designated. The Principal Investigator commits to carry out the study in compliance with the protocol, amendments, applicable procedures and other study-related documents provided by the Sponsor, and in compliance with applicable ethical and regulatory requirements.

The protocol and all relevant information will be made available to all relevant government institutions of Mali who participate in conducting this study.

This document may contain information that is privileged or confidential. As such, it may not be disclosed to any other than involved research staff, research partner institutions and the concerned Ethics Committee(s), unless specific permission is granted in writing by the International Food Policy Research Institute, or such disclosure is required by federal or other laws or regulations. In any event, persons to whom the information is disclosed must be informed that the information is privileged and confidential and may not be further disclosed by them.

Principal Investigator International Food Policy Research Institute

Name: Dr Marie Ruel   Date: 6 October 2014

Signed:

Principal Investigator International Food Policy Research Institute

Name: Dr Rahul Rawat   Date: 6 October 2014

Signed:

Principal Investigator Helen Keller International

Name: Dr Lazare Coulibaly   Date: 6 October 2014

Signed:

Table of Contents

1. PROBLEM STATEMENT AND STUDY JUSTIFICATION 1

2. OBJECTIVES, RESEARCH QUESTIONS AND HYPOTHESES 3

2.1 OVERALL OBJECTIVE 3
2.2 RESEARCH QUESTIONS 4
2.3 RESEARCH HYPOTHESES 4

3 RESEARCH METHODS 5

3.1 STUDY POPULATION 5
3.2 PROGRAMME DESCRIPTION 6
3.2.1 GENERAL INFORMATION ON THE NATIONAL CMAM PROGRAMME 6
3.2.2 CMAM HKI ACTIVITIES IMPLEMENTED FOR PROMIS IN THE BLA AND SAN HEALTH DISTRICTS... 8
3.3 IFPRI IMPACT EVALUATION 10
3.3.1 STUDY DESIGN 10
3.3.2 RANDOMIZATION 11
3.3 MEASUREMENT AND PROCEDURES 15
3.4 DATA MANAGEMENT AND ANALYSIS 18

4. ETHICAL APPROVAL 19

5. PARTNERSHIP AND MANAGEMENT 20

6. BUDGET 21

7. TIMELINE 22

8. REFERENCE LIST 22

List of Abbreviations

ABC	Activity Based Costing
BCC	Behavior change communication
CHA	Community Health Associations
CHD	Child Health Days
CHV	Community health volunteers
CHW	Community Health Workers
CMAM	Community-based Management of Acute Malnutrition
CSCom	Centres de Santé Communautaire
CSref	Centres de Santé de rérérence or Reference Health Centers
DFATD	Department of Foreign Affairs, Trade and Development
DHT	District Health team
DMO	District Medical Officer
ENA	Essential Nutrition Actions
FGD	Focus Group Discussion
HAZ	Height-for-Age Z-score
Hb	Hemoglobin
HKI	Helen Keller International
IFPRI	International Food Policy Research Institute
IYCFI	Infant and Young Child Feeding Indicators
MAM	Moderate Acute Malnutrition
MNCH	Maternal, Newborn, and Child Health
MNP	Multiple micronutrient powder
MoH	Ministry Of Health
MUAC	Mid-Upper Arm Circumference
NGO	Non-Governmental Organization
ORS	Oral Rehydration Salts
RUSF	Ready-to-Use Supplementary Food
RUTF	Ready-to-Use Therapeutic Food
SAM	Severe Acute Malnutrition
SQ-LNS	Small Quantity Lipid Based Nutrient Supplement
UNICEF	United Nations Children's Fund
USAID	United States Agency for International Development
VAS	Vitamin A Supplements
WASH	Water, Sanitation and Hygiene
WHO	World Health Organization
WHZ	Weight-for-Height Z-Score
WFP	Word Food Programme

1. Problem statement and study justification

Globally, child undernutrition is underlying cause for 3.1 million deaths of children younger than 5 years, representing about 45% of all deaths in this group. Within this context, 18.7 million children under five years of age suffer from severe acute malnutrition (SAM), defined by a Weight-for-Length Z-score less than -3 or a MUAC<115 mm. An additional 33 million children under five are at risk of becoming SAM being in a stage of moderate acute malnutrition (MAM, defined by a WHZ<-2 or a MUAC <115mm). This situation is aggravated by the presence of child chronic malnutrition expressed by stunted linear growth that affects 163 million children worldwide and important micronutrient deficiencies of which the most problematic are vitamin A, zinc, iron and iodine [1].

The landscape for treatment of child severe acute malnutrition has changed dramatically in the last 10 years with the advent of ready-to-use therapeutic foods (RUTFs) that are easier to manage in field conditions, and the adoption of treatment protocols that promote treatment of cases of SAM without complications in outpatient programmes supported by community-based and primary health care delivery platforms through an approach called Community-based Management of Acute Malnutrition (CMAM).

However, in contrast to an isolated management of SAM, the CMAM model consists of an integrated approach for the management of both SAM and MAM. In 2012, the WHO formulated a set of recommendations for supplementary feeding programs specifically aimed at MAM that are currently being implemented in 89 countries worldwide under the umbrella of CMAM[2]. Access to services for treatment of Acute Malnutrition (AM), i.e. MAM and SAM, has expanded rapidly and has proven to be highly effective for saving the lives of acutely malnourished children.

In countries like Mali that have increased availability AM services, a major challenge remains in ongoing, effective screening and referral coverage to optimize early detection and uptake of these services. A recent survey in Mali quantified screening coverage of child acute malnutrition by bi-annual mass-screening campaigns (Child Health Days), community health volunteers or health center staff [3]. Whereas participation in Child Health Days was quite high (82% of 1543 eligible children), only 52% of the participating children were screened using the MUAC criterion. Routine screening performed even worse with only 22% and less than 5% of eligible children screened by community health volunteers and health center staff respectively. An even more worrisome finding was that only 57% of the screened cases during the CHD actually visited a health center for a confirmatory diagnosis and the initiation of treatment.

Preventing all forms of child undernutrition requires ensuring optimal infant and young child feeding practices and preventing and treating infectious diseases. Exclusive breastfeeding is recommended for the first 6 months of a child’s life, after

which complementary foods are needed to complement breast milk and ensure adequate intake of essential nutrients. A recent summary of the rather limited evidence on the effect of child nutrition education or BCC pointed at modest, but statistically significant, effect size on linear growth (0.22; 95%CI: 0.01-0.43), however most of the studies were conducted in food secure settings [4]. In most resource-constrained environments, especially in Sub-Saharan Africa, households lack the means to ensure a nutritionally adequate diet for their 6–23 month old children; hence a more limited impact by BCC interventions only on child undernutrition should be expected. The advent of specialized products, however, such as multiple micronutrient powders (MNPs) and small quantity lipid-based nutrient supplements (SQ-LNS) provides an opportunity to combine behavior change communication (BCC) programs that promote optimal Essential Nutrition Actions (ENA) and Infant and Young Child Feeding (IYCF) practices with access to products that help food-insecure households provide a nutritionally adequate complementary feeding diet to their young children. To date, the evidence on the efficacy of MNP and LNS on child undernutrition is not completely consistent. A recent Cochrane review concluded that MNPs were effective in cutting the risk of iron deficiency in half, but effects on vitamin A and zinc levels were less consistent between studies[5]. Interestingly though, in contrast to MNPs[4–6], embedding the multiple micronutrients in a small quantity of energy dense spread like LNS triggered additional effects on linear growth and the prevention of stunting in several intervention studies.[7–9]

There is often poor integration between programs to treat child acute malnutrition and programs focused on the prevention of acute and chronic undernutrition – resulting in many missed opportunities for using prevention platforms to screen and refer SAM children, or for using screening and referral platforms to provide prevention services. Reasons for this discrepancy include the different actors or delivery channels of treatment and prevention of child undernutrition and the different dynamic of these programs or strategies. Whereas the treatment of acute malnutrition evolved from an emergency response and is defined a more limited time frame, preventive BCC interventions might require a longer time frame. In resource-limited settings, a combination of health facility and community-based platforms seems a promising strategy to harmonize both preventive and curative interventions. Employing community-based platforms with lay health workers to promote maternal and child health and development typically results in improved immunization coverage, better breastfeeding practices and reduced child morbidity and mortality compared to usual care.[10]

Effective BCC combined with the distribution of specialized products such as RUTF for SAM, RUSF for MAM, and SQ-LNS for the prevention of both acute and chronic undernutrition are key elements of successful programs to tackle the whole spectrum of childhood undernutrition problems – from prevention of chronic undernutrition to prevention and treatment of AM. A variety of program models combining BCC and specialized products have been developed for different

contexts, but preventive and treatment programs have generally been implemented in a parallel rather than in an integrated manner.

This project will address two critical gaps related to the integration of preventive and treatment programs: 1) screening and treatment of AM have not yet been systematically integrated into routine health-center visits or mainstreamed into community outreach programs; and 2) screening programs do not offer any preventive services for those children found not to be suffering from AM at the time of screening; mothers of children identified as non-acutely malnourished case are usually sent home without receiving any health or nutrition inputs and as a result, may fail to come back for screening because they do not see any tangible benefit associated with their participation in the screening. This project will specifically address these gaps by identifying and testing innovative platforms to integrate treatment and prevention interventions for both chronic and acute malnutrition.

2. Objectives, Research Questions and Hypotheses

2.1 Overall objective

The overall study objective is to reduce the prevalence of child malnutrition by integrating preventive and treatment protocols through combined health facility and community based platforms.

This study aims at delivering empirical evidence that the CMAM program implemented by HKI which integrates preventive and treatment schedules related to child undernutrition, when combined with the provision of SQ-LNS through community-based systems, is able to improve child nutritional status and infant and IYCF knowledge and practices.

In particular, the HKI program combines i) enhanced case-finding, referral and compliance to treatment; ii) strong preventive component combining the distribution of a small quantity of LNS and a strengthened nutrition education component - the HKI Essential Nutrition Actions (ENA) framework. Moreover, a strong focus will be put on the community-based platform of health workers and volunteers that already provide basic BCC related to child nutrition and health.

2.2 Research Questions

A rigorous program evaluation of HKI's program will be performed by IFPRI. For evaluations purposes, the following research questions will be addressed:

I. Feasibility of integrating preventive services into treatment programs

1) Is provision of SQ-LNS through village-based BCC gatherings logistically feasible?
2) Is provision of enhanced IYCF/ENA/WASH BCC through village-based monthly meetings logistically feasible?
3) Is the SQ-LNS product culturally acceptable and used as recommended for the prevention of child undernutrition?

II. Impacts on proportion of children screened for AM (SAM and MAM cases), uptake and completion of treatment

1) Does provision through village-based monthly gatherings of enhanced IYCF/ENA/WASH BCC and SQ-LNS distribution to 6-23 mo old children increase:
   a. Proportion of children screened for AM?
   b. AM outpatient treatment compliance and rate of completion of full treatment?

III. Impacts on incidence and prevalence of acute malnutrition

1) Does provision of enhanced IYCF/ENA/WASH BCC and SQ-LNS to 6-23 mo old children reduce:
   a. the incidence of wasting (new cases of AM)
   b. the prevalence of wasting (through prevention and improved screening/referral/treatment) at study termination?
2) Does provision of enhanced IYCF/ENA/WASH BCC and SQ-LNS to 6-23 mo old children reduce:
   a. the incidence of child stunting (HAZ<-2)?
   b. the prevalence of stunting at study termination?
3) Does the provision of SQ-LNS and BCC through village-based monthly gatherings result in an improved behavior by the caregiver, related to child's health and nutrition including improved IYCF practices?
4) What is the cost and cost effectiveness of providing preventive SQ-LNS and monthly BCC through village-based monthly gatherings on screening, prevention and treatment of child undernutrition?

2.3 Research hypotheses

In comparison with a control group consisting of a sample of children 6-23 months of age from health center catchment areas where monthly BCC related to ENA/IYCF/WASH is provided, monthly distribution of SQ-LNS by community workers (CHW, CHV):

1) Reduces the prevalence of AM in children 6-23 months of age
2) Reduces the incidence of acute malnutrition (new cases of MAM) in children 6-23 months of age
3) Reduces the prevalence of chronic malnutrition (stunting) in children 6-23 months of age
4) Reduces the incidence of new cases of stunting in children 6-23 months of age
5) Increases proportion of children screened for AM (defined as the number of study children screened in the month preceding the survey over the total number of eligible study children)
6) Increases AM treatment compliance (defined as the number of AM children who received appropriate treatment in the month preceding the survey over the total number of AM cases identified in the study sample)
7) Increases blood hemoglobin concentration in children 6-23 months of age
8) Reduces anemia (Hb<11g.dL^{-1}) in children 6-23 months of age
9) Improves IYCF/ENA/WASH knowledge and practices
10) Results in a better cost-effectiveness to reduce the prevalence of acute and chronic malnutrition in children 6-23 months of age.

3 Research Methods

3.1 Study population

Mali is a low-income, West African country with nearly 13 million inhabitants, 77% of whom live on less than US$2/day. The study will be conducted in the health district of San and Bla situated in the Ségou region that includes 58 Health Centers (HC) and two reference HC. Population statistics of these districts show a total of 146,700 children aged 6-59 months. From the most recent Demographic Health Survey (2012-2013) a 12.9% prevalence of child acute malnutrition together with a 40.5% prevalence of child chronic malnutrition or stunting was reported for the Ségou region [11]. During the last 5 years, Helen Keller international has supported the implementation of integrated CMAM to tackle the burden of child acute malnutrition by improved screening, referral and treatment of acute malnutrition. In 2013, a coverage survey conducted by HKI in the Koutiala district of the neighboring Sikasso region estimated that the CMAM coverage was only 25.7% [12]. The main barriers to better coverage included lack of awareness of the problem of child malnutrition, insufficient routine case-finding (screening), low quality of care at health centers and stock-outs of therapeutic nutritional products. In addition, interviews with non-participating beneficiaries pointed at an existing

stigma of child malnutrition in this population hindering spontaneous participation and referral for treatment of positive cases of acute malnutrition.

3.2 Programme Description

3.2.1 General information on the national CMAM programme

3.2.1.1 National CMAM strategy

At the community level, health services are provided by health centers. HCs are supposed to be staffed by a physician, nurse, trained birth attendants and supported by CHW and CHVs.
Since 2010, the MOH has followed the task shifting strategy of allowing provision of *Soins essentiels dans la communauté* or SEC (community level essential health care) by CHWs. These services include the integrated community case management of childhood illness (IMCI: malaria, pneumonia, diarrhea) and acute malnutrition, as well as family planning, essential newborn care, and hygiene and sanitation. However, for now, only 60% of the HC catchment areas in Bla and San is covered by CHWs.
CHWs are authorized to provide outpatient treatment to both SAM and MAM cases at the community level as well as CSCom staff. In-patient care is organized at the nearest CSRef (Centres de Santé de rérérence or Reference Health centers). CHVs help follow-up children diagnosed with MAM or SAM to support their completion of treatment and improved nutrition practices. Since the crisis precipitated by the overthrow of the previous government, the supply of Plumpy'Nut (RUTF) for SAM and Supplementary Plumpy (RUSF) for MAM have been steady, and delivery to health districts assured by UN agencies. Delivery to CSComs can be more problematic, as cannot accurately project their needs.

Behavior change communications (BCC) for health is led by the MOH but also supported by the Ministry of Social Development (*Ministère du Développement Social et de l'Economie Solidaire*), which assigns around 2-3 social development agents (*Agents de Développement Social*; ADS) to each district to shape the communication strategy of towards communities.

3.2.1.2 Screening platforms

Mali approved an update of the CMAM protocol in 2012, which uses the WHO admission and discharge criteria for the treatment of child acute malnutrition. **Routine screening for acute malnutrition takes place at the CSCom** during 1-

2 screening days each week (and theoretically during each sick and well child consultation). Health staff uses either MUAC tapes or weight for height measures for diagnosis. There is also a **biannual child mass screening** event organized at the community level called SIAN (*Semaine d'Intensification des Activités de Nutrition*) with donor support and carried out by CSCom staff and CHVs. In addition, CHWs and CHVS can organize **monthly screening events at the community level;** this also depends largely on external support. Mass and community-based screening is done with MUAC measures.

To date there is no consistent organization linking these parties to obtain maximal coverage of AM screening. In villages screening happens mainly on demand of the caregiver and coverage is expected to be rather low. Biannual SIAN campaigns typically result in good coverage, however, SIAN are only organized twice a year, which hampers early detection of many AM cases and increases case-fatality rates as a consequence.

3.2.1.3 Referral and treatment schedules for MAM and SAM

When community workers screen a child for acute malnutrition, he is either diagnosed as negative (green code), positive MAM (115mm<MUAC<125mm; yellow code) or positive SAM (MUAC<115mm or bilateral pitting edema; red code). In the case of SAM, mother-child pairs are referred to the HC for a confirmatory diagnosis based on MUAC or Weight-for-Height (WH) criterion and to initiate treatment. The mother receives a referral slip (red color) with the MUAC value or edema observation. The community worker does not write down the name of the person but annotates the number of cases referred monthly. For MAM cases, the referral procedure depends on the type of village. For villages nearby a HC, cases are referred to the HC carrying a yellow referral slip with the MUAC/edema findings. For more remote villages MAM cases are referred to a CHW that provides outreach services that will confirm the diagnosis using the MUAC criterion only.
In case of a confirmatory diagnosis of SAM, the child is examined further for medical complications. In the absence of such signs and after a successful appetite test, the child is included in an outpatient program with a weekly dose of RUTF (Plumpy Nut®, Nutriset, France). The mother receives a referral slip with the HC’s diagnosis destined for the CHW or CHV that ensures a follow-up role in the village. Complicated cases of SAM or with clear signs of edema are referred for inpatient treatment to the hospital (CSRef) that disposes of a pediatric unit.
In case of a confirmatory diagnosis of MAM by a CHW based on the MUAC criterion (MUAC or by the HC staff based on MUAC and WH criterion), the mother receives a supplementary food ration (CSB++ or Supplementary Plumpy) and is followed-up every week.

3.2.1.4 Nutrition education

Currently, nutritional education group sessions are organized at the HC level before immunization sessions 1-2 times each week. These sessions are focusing on delivering general messages about optimal breastfeeding practices and the preparation and introduction of complementary foods. In addition, most villages have 1-3 CHV who promote preventive practices such as nutrition BCC, hand washing, household hygiene, diarrhea treatment with oral rehydration salts, antenatal care, family planning, and the prevention of malaria. Community engagement around health is strong, and many volunteers are active in promoting well-being among their neighbors. Women, men, youth, and community groups, also support health needs through savings/credit clubs and professional organizations. However, to date, a solid evaluation on the effectiveness and the coverage of this promotion is lacking.

3.2.2 CMAM HKI activities implemented for PROMIS in the Bla and San Health Districts

HKI supports the national CMAM strategy that is inscribed in the National Nutrition policy plan. For PROMIS, HKI general activities will include the continuation and expansion of the approaches outlined above, with focusing on the following tasks:

- **Capacity strengthening and formative supervision to improve quality of screening, referral and curative care:**
  HKI will follow the updated 2012 National CMAM protocol validated by nutrition partners through the Nutrition Cluster and by the Ministry of Health Nutrition Division to train currently untrained health workers (CSref/CSCOM staff and CHW) and CHV. Refresher training will be foreseen for previously formed staff and CHW. In addition, HKI will organize formative supervision in CSComs, CHWs and CHV by project staff together with the MoH. Specific attention will be dedicated to the quality of acute malnutrition screening at the health facility level, during IMCI, and by CHW/CHV in the communities. In addition, HKI will introduce a solid system of communication between actors who conduct screening, referral, treatment and follow-up. Specific supportive actions from HKI include:

- **Support to the MoH to improve coverage and quality of SIAN** related to nutrition activities and screening for acute malnutrition. HKI will support the MoH to organize the biannual child health days (SIAN). In addition to the acute malnutrition screening of children of 6 to 59 months of age, typical activities include vitamin A supplementation for children 6–59 months of age and for early post-partum women and deworming of children 12–59 months.

- **Capacity building to improve quality of BCC**:

- HKI has been developing from many years the ENA framework as a BCC tool, which encompass 7 actions areas (or messages): 1. Women's Nutrition; 2. Breastfeeding; 3. Complementary feeding; 4. Feeding of sick children; 5. Integrated Control of Anemia; 6. Control of Vitamin A deficiency and Control of Iodine Deficiency Disorders. In addition, HKI is developing the Essential Hygiene Actions, which will be integrated within HKI BCC.

- HKI will strengthen CSCom staff, CHW, and CHV's capacities on disseminating ENA messages, in particular those targeted to infants (related to breastfeeding and complementary feeding practices, or IYCF practices).

- The project will support community ownership and empowerment on BCC messages by introducing negotiation skills (SBCC=Social and Behavior Change Communication) to promote the adoption of new behavior related to nutrition, health and hygiene among households with children under 5 years of age and analysis of main barriers for adoption by the community themselves.
- CHV will visits caregivers at home of young children (0-23 months) to invite them to **monthly group counseling sessions**. These sessions will support families in overcoming barriers to new child nutrition and health related practices. These sessions will be led by CSref/CSCom health staff for villages near to a CSref/CSCom and by CHW for more remote villages. For villages with no CHW, CHV will take the lead in organizing these group sessions and provide BCC.
- Broadcasts from local radios and local advocacy efforts will further reinforce essential messages. Engaging Community health management committees (ASACOs) and community leaders will be a key factor to ensure sustainable preventive and CMAM services. In addition, community events will be organized around WASH and nutrition themes, including hand washing days.

- Reinforcement of logistics management to strengthen the supply chain of supplementary foods and RUTFs for the treatment of MAM and SAM respectively. More precisely, HKI will support the district and CSCom level to communicate better estimations of the required quantities to UNICEF and WFP to reduce stock-out of these nutritional food supplies.

- Distribution of SQ-LNS:
  SQ-LNS will be distributed at monthly group counseling sessions. During these sessions caregivers of children 6-23 months old will be provided with a monthly dose (considering an average daily doses of 20g) and will be

sensitize to the adequate use of SQ-LNS to situate the SQ-LNS in the overall child's diet.

During these BCC sessions, participating children will also be screened for acute malnutrition and referred to HC or CHW for treatment if needed. Such integration between BCC and screening is expected to have a synergetic impact on the reduction of acute malnutrition prevalence by preventing new cases of AM (MAM and SAM) and by ensuring better screening, referral and treatment rates of those cases. Indeed, also AM cases will continue to participate in these group-counseling sessions allowing CHW or CHV to follow-up on their treatment schedule.

Monthly SQ-LNS distribution, as part of the delivery of the preventive package, will be conducted by health workers at HCs for nearby villages as well as through community structures by CHW and CHV. In villages that are more remote from HCs and where CHW are present, CHWs will organize the SQ-LNS distribution in collaboration with CHV. In villages without CHW, CHV will ensure the distribution in close collaboration with HC staff of that catchment area.

3.3 IFPRI impact evaluation

The evaluation of the program model in the Bla and San districts will compare two groups:

1) The intervention group receiving current reinforced CMAM program with BCC related to ENA, IYCFI and WASH, and distribution of SQ-LNS with sensitization for the adequate consume of SQ-LNS as a preventive measure to children 6-23 months of age.
2) The control group will receive current reinforced CMAM program with BCC related to ENA, IYCFI and WASH.

3.3.1 Study design

The study design entails a two-arm stratified cluster-randomized, non-masked, community-based, effectiveness trial. The unit of randomization will be the catchment area of a health center.

Both arms will benefit from a reinforced CMAM program combined with monthly group BCC counseling sessions. Subjects in the intervention arm will receive additional monthly SQ-LNS and LNS-use sensitization.

Because of the intended dual role of BCC/SQ-LNS on child undernutrition in this study – e.g. to help prevent child undernutrition and enhance the proportion of children screened, referral and treatment of AM, it is necessary to combine two study designs to rigorously evaluate the impact of the proposed intervention and to tease out the contribution of prevention and enhanced coverage/treatment to the overall impact on child malnutrition.

The proposed study will therefore use two types of study designs. The first one is a **repeated cross-sectional design** that will compare selected study outcomes between intervention and control groups at endline, after 24 months of program implementation. A repeated cross-sectional study design among children 6-23 months, at baseline and after 24 months (on different children) will be used to assess the impact of the intervention on the *prevalence* of several outcomes, including the prevalence of AM and stunting, the proportion of children screened for AM and maternal ENA/IYCF/WASH knowledge and practices. The second proposed study design entails a **longitudinal design** whereby individual children will be recruited at 6 months of age and followed-up monthly until they reach 24 months of age. We anticipate needing approximately 3 months to recruit the required number of children. This design will allow us to assess the intervention's effects on the *incidence*, recovery and recurrence rates of AM cases (MAM and SAM).

3.3.2 Randomization

Because of the substantial variation in the efficiency and functioning of the health centers and the attached community extension network we will first stratify the health centers by non-hierarchical clustering using a set of criteria that are enlisted in Table 1. Stratifying clusters prior to randomization is a recommended practice to ensure a more balanced distribution of cluster-level covariates between study arms. Based on the obtained cluster dendrogram we will subdivide the clusters into 3-4 strata after which random allocation to control or intervention groups will be conducted for every stratum. The study area encompasses 48 of the 58 catchment areas of health centers in the Bla and San health districts situated in the Ségou region in the East of Mali. Random allocation of these 48 clusters to intervention and control arms will be conducted during a community ceremony in the presence of local health authorities. Small identical papers with the cluster names will be mixed in a bag and a volunteer will draw one paper at a time. The allocation will alternate between both study groups. The first draw will be allocated to the control group, the next to the intervention group, etc.

Table 1: Criteria to stratify clusters

Criteria
- Type of staff working in HC
- Accessibility during rainy season
- Type of the Catchment area (urban/rural)
- Number of villages
- Number of villages with CHW
- Vaccination coverage
- Total number of children 6-23 months
- Proportion MAM admissions/total population
- Proportion SAM admissions/total population
- Distance between villages and HC

- Distance between HC and Reference HC (CSRef)

3.3.2.1 Repeated cross-sectional study

- *Study outcomes*

The first primary study outcome is the prevalence of child AM at endline. An anticipated lower AM prevalence after program completion could be the result of either a successful prevention of MAM (less new cases) or a better case-finding, referral and treatment of MAM and SAM or a combination of both.

The second primary outcome is the prevalence of child chronic malnutrition or stunting at study endline. Typically, in Sub-Sahara African and Asian populations, linear growth retardation starts at from conception and continues to the age of 24 months after which growth stabilizes.[13] These first 1,000 days of life are also called the window of opportunity for nutrition interventions, because it is believed that during this period linear growth retardation can be prevented. On average, the most dramatic deceleration in linear growth velocity happens between 6 and 24 months of age. Optimal linear growth is the result of a great many determinants, amongst which appropriate complementary foods and breastfeeding, good hygienic practices, food safety and disease prevention. For this purpose, it is an appropriate outcome to evaluate an intervention program that provides BCC aimed at improving IYCF, ENA and WASH practices in combination with a home-fortificant like SQ-LNS.

Primary study outcomes include:

- Prevalence of global acute malnutrition (AM) defined by WHZ<-2 or MUAC[1]<125mm at study endline in children 6-23 months of age
- Proportion of children screened for AM (defined as the number of study children screened in the month preceding the survey over the total number of children 6-23 months of age)
- AM treatment compliance (number of AM children who received treatment in the month preceding the survey over the total number of AM cases identified in the study sample)

Secondary study outcomes include:

- Prevalence of child stunting defined by HAZ<-2 for children 6-23 months of age.
- Mean HAZ-score at endline for children 6-23 months of age;
- Mean height at endline for children 6-23 months of age;

[1] Throughout the study, MUAC will only be used among children aged ≥ 6 mo

- Mean WHZ-score at endline for children 6-23 months of age;
- Mean MUAC at endline in children 6-23 months of age;
- Mean hemoglobin concentration at endline in children 6-23 months of age;
- Prevalence of child anemia (Hb concentration<11g.dL^{-1}) at endline in children 6-23 months of age;
- Prevalence of SAM defined by a WHZ<-3 or bilateral pitting edema or a MUAC<115mm
- Prevalence of severe stunting defined by a HAZ<-3
- Caregiver's knowledge of indicators of IYCF, ENA and WASH
- Caregiver's practices related to IYCF, ENA and WASH

- *Study size*

In order to detect a 30% decrease in AM prevalence (assuming 13.5% AM prevalence at baseline) with a statistical power of 80%, assuming a type I error of 5%, a n ICC of 0.01 and a non-response rate of 15%, a total number of ≈48 clusters with an average cluster size of 48 children is required, which results in a total sample size of 2,304 children – 1,152 for the control arm and 1,152 for the intervention arm.

- *Sampling strategy and study enrollment*

Between December 2014 and February 2015, a first cross-sectional study will be organized before the program is implemented to document the pre-intervention situation. An identical endline cross-sectional study will be conducted during the same months in 2016-2017 to counter any monthly seasonal variations. From every health center catchment area, 2-4 villages will be selected with a probability proportionate to their population size in terms of children 6-23 months of age. Thereafter, 48 households with at least one index child fulfilling the inclusion criteria will be randomly selected from a census list of selected villages per health center catchment area.

Inclusion criteria for the cross-sectional surveys are:
- At least one index child 6-23 months of age in the household
- Mother should be living in the study area since the index child's delivery
- Singleton infants

Exclusion criteria for the cross-sectional surveys are:
- Index child should not present congenital deformations that hamper anthropometric measurements

Age is an important covariate for anthropometry and IYCFI, therefore we opt to stratify the sampling by age to ensure comparable samples from the control group and the intervention group

3.3.2.2 Longitudinal study

- *Study outcomes*

Primary study outcomes include:

- Incidence of AM defined by WHZ<-2 or MUAC<125mm or bilateral pitting edema in children followed-up monthly from 6 to 24 months of age
- Proportion of children screened monthly (number of study children screened in the month preceding the home visit over the total number of eligible study children) over the total 18 months' follow-up period
- Compliance to treatment of acute malnutrition (number of AM children adhering to treatment over the total number of AM children enrolled for treatment) over the total 18 months' follow-up period.

Secondary study outcomes include:

- Incidence of child stunting defined by HAZ<-2 in children followed-up monthly from 6 to 24 months of age
- Longitudinal prevalence of AM, MAM and SAM defined by time the child was acutely malnourished over the total follow-up time
- Change in mean AM prevalence over time
- Treatment compliance of MAM and SAM, enrollment, recovery of AM
- Relapse rate after treatment of AM (%WHZ<-2 or MUAC<125mm or bilateral pitting edema after discharge from MAM or SAM treatment program)
- Mean episode length for AM, MAM and SAM
- Linear growth velocity (HAZ increment/month)
- Ponderal growth velocity (WHZ increment/month)
- Weight gain (weight increment/month)
- MUAC gain (MUAC increment/month)
- Change mean HAZ over time
- Change mean WHZ over time
- Change mean MUAC over time
- Infant morbidity: acute respiratory infections, fever, diarrhea, vomiting and malaria (longitudinal prevalence)

- *Study Size*

To ensure maximal comparability between results of the repeated cross-sectional study and the longitudinal study we opt to work in the same 48 clusters of the Bla and San districts. Randomizing 48 clusters with an average cluster size of 23, assuming an ICC of 0.022 and a dropout rate of 20% would allow to detect a 26.3% difference in AM incidence over a follow-up period of 18 months (from 6 to 24 months of age) with a statistical power of 90% and a type I error of 5%.[14] Hence

the total sample size for the longitudinal study is 1,104 or 552 for the control group and 552 for the intervention group.

- *Sampling strategy and study enrollment*

From April to June 2015 children 6-6.9 months of age and fulfilling the study's inclusion criteria will be enrolled in the cohort. For this purpose mass gatherings will be organized in every village of the study area. Community workers will be asked to gather all children of that age to be screened for study eligibility.

Inclusion criteria for children for the longitudinal study are:

- 6-6.9 months of age;
- Child with WHZ>-2 and MUAC>125 mm and no bilateral pitting edema
- Mother should be living in the study area since the index child's delivery
- Singleton infants

Exclusion criteria include:

- Congenital malformations that make anthropometric measurements impossible
- Mother planning to leave the study are in the coming year

3.3 Measurement and Procedures

Questionnaires

- Health center level: A module with questions will be administered to the health center's staff. Questions on past, current and future projects related to health and nutrition in the project area will be asked. Interviewers will conduct a facility assessment enlisting available protocols related to CMAM, but also any preventive strategies on child malnutrition. Furthermore, available health statistics from the health information system will be annotated. Time allocation of staff to CMAM at health center and during outreach activities, overall job motivation and job satisfaction will be documented. Finally, perceived barriers and boosters on CMAM treatment will be recorded.

- CHW/CHV level: a model with questions on ENA/IYCF/WASH knowledge, time allocation, motivation, job supervision, and job satisfaction will be administered. Additionally, specific questions will focus on the screening, referral and follow-up of AM cases under treatment in the outpatient CMAM part. Furthermore, questions on previous participation in BCC will be formulated.

- Household level: Questionnaires on household composition, socio-economic status, household expenditure, household food security, cattle/land/asset ownership and construction materials of the concession will be administered. In addition, there will be a module of questions on food hygiene, hygienic practices and sanitation. Furthermore, household food security and participation in social or NGO or government-related programmes will be assessed.

- Caregiver and child level: Questions on father and mother's education level, primary profession, decision power over household budget and the index child's health and nutrition will be administered. Furthermore, specific questions on ENA/IYCFI/WASH knowledge and practices will be asked to the child's primary caregiver. Further topics include utilization of health care services, contact moments with CHW and CHV. Child's age will be obtained from a birth certificate or using a locally adapted event calendar in the case an objective source of date of birth is lacking.

Anthropometry

The child's weight will be recorded using an electronic scale (SECA 876, Germany) to the nearest 100g. Length will be recorded to the nearest 1 mm using a length board (SECA 417, Germany). Mid-upper arm circumference will be recorded by using non-stretchable tape with 0.1cm precision (SECA 201, Germany). All measurements will be taken in duplicate by an anthropometrist and an assistant. All measurements will be exercised before the study through standardization exercises. Standardization exercises recording measurements from 10 children between 6-23 months of age will be repeated bimonthly. From these standardization sessions inter- and intra-observer variation of measurement error will be documented. Maternal MUAC, weight and height will also be recorded using non-stretchable tape (SECA 201), scales (SECA 877) and stadiometers respectively. WHZ and HAZ scores will be calculated using the 2006 WHO growth reference.

Morbidity and mortality in the longitudinal study

Data collectors from the evaluation team will conduct monthly home visits to recall infant morbidity (Acute Respiratory Infections, Diarrhea, Fever and Malaria) of the last 3 days. A diarrheal episode is defined by at least 3 liquid stools in the last 24h, or stool with blood. Fever will be recorded by a standard thermometer, but will also be recalled. The presence of acute respiratory infection (ARI) during the previous week will be assessed by recalling specific ARI-related symptoms (cough, difficult breathing, grunting, rapid breathing, and nasal secretion). Malaria will be diagnosed with a CareStart™ Malaria Pf/Pv Combo rapid diagnostic test by taking

finger blood from the child with a lancet in case a body temperature of more than 37.5°C is measured or if the mother reports the occurrence of a child fever episode over the last 12 hours. Using a 3-day recall for morbidity often leads to only a small to moderate loss of statistical power compared with a 7-day recall period, especially if morbidity signs are common, if the number of measurements per individual exceeds 10 or 12 and in cluster randomized trials.[15]
Morbidity outcomes will be expressed as a longitudinal prevalence by taking the proportion of the days with recalled symptoms over the observed period in days.

Hemoglobin in capillary blood

Hemoglobin concentration will be measured at baseline and endline of programme. For this purpose finger blood will be sampled. Hemoglobin concentration will be measured by spectrophotometry using a HemoCue device (HemoCue Ltd, Dronfield, United Kingdom). The device will be calibrated on a daily basis using a HemoCue Control Cuvette.
Child anemia and severe anemia is defined by a hemoglobin concentration less than 11g.dL^{-1} and less than 7 g.dL^{-1} respectively.

Cost and cost-effectiveness

Costs will be estimated from the societal perspective, thereby including all relevant costs incurred by institutions and communities.[16] Costs will be calculated with a combination of accounting records and "ingredients" estimates using unit costs and quantities of inputs.[17] An activity-based costing (ABC) methodology will be employed, allowing categorization and allocation of all costs of the program to its primary activities. Financial costs as well as economic costs (in-kind donations, household costs, etc.) will be collected. To obtain this data, program documentation and expenditure information will be reviewed, and key informant interviews and focus group discussions (FGDs) will be conducted with relevant program implementation staff. Another output of these interviews and FGDs will be time allocation estimates; staff time allocation will be used according to ABC methodology to allocate personnel costs among activities. FGDs will be conducted with households to gain an understanding of the direct and indirect costs they incur while participating in the program. The effect of plausible variation in household cost estimates from FGDs will be assessed during sensitivity analysis.
Analysis will focus on both total program costs, and incremental costs of the additional activities in the intervention area. Cost Effectiveness ratios (CER) will be calculated using costs and outcomes of the program. Incremental cost-effectiveness ratios (ICER) will be calculated by dividing the additional costs in the intervention area by the number of cases of acute malnutrition averted in the intervention area compared to the control area. This ratio represents the additional cost to achieve an additional successful outcome in the intervention area relative

to the control area. Comparison with other similar published works will be considered where appropriate.

All aforementioned measurements except for the cost-data will be part of the cross-sectional surveys. For the longitudinal study, monthly anthropometry, child morbidity, questions on age-specific IYCF/ENA/WASH practices will be included as well as a monthly recall of participation in either mass screening campaigns during SIAN, routine screening by HC, CHW or CHV or screening during monthly PROMIS counseling sessions. A separate module of questions related to referral and treatment will be foreseen in case a child was found acutely malnourished.

3.4 Data management and analysis

Enumerators will use small laptops with Computer Assisted Personal Interviews (CAPI) for this survey work (SurveyBe software).

Baseline data from the cross-sectional survey will be used to assess the quality of the cluster randomization and document the pre-intervention situation. For this purpose, cluster and individual level means and standard deviations will be presented if variables are normally distributed. Medians and interquartile distances will be given in case continuous variables do not follow a normal distribution. Proportions will be presented for categorical variables.

The intervention effect on outcomes from the repeated cross-sectional study will be analyzed using the double difference specification that calculates the difference between endline outcomes in the control and intervention arm and subtracts the difference between baseline outcomes in those same arms to construct an estimate of program impact. Although the randomization is expected to minimize average differences at baseline between groups, this approach accounts for any remaining small differences at baseline, improving the precision of estimates. Formally, estimating double-difference impacts translates to running the Ordinary Least Squares (OLS) regression shown in equation 1.

$$Y = \beta_0 + \beta_1 * \text{Intervention} + \beta_2 * \text{Time} + \beta_3 * \text{Intervention} * \text{Time} + \varepsilon \quad \text{(eq. 1)}$$

With *Intervention* being a binary variable designating either control or intervention group, *Time* being a binary variable that either designates the baseline or endline survey time point. The double difference is then given by the β_3 coefficient.
These regression models will further be adjusted for clustering at health center and village level. Furthermore, the analysis will be adjusted for covariates that differ more than 5% at baseline. Linear and binary logistic regression models will be used for continuous and binary outcomes respectively.

Ponderal (weight, MUAC and WHZ increments) and linear growth (length and HAZ increments) obtained from the longitudinal study, will be modeled using mixed-

effects model with restricted splines with knots at 6, 12, 18 and 24 months of child age. Covariates include the intervention group allocation, linear and spline terms for time (in months), and interaction terms between intervention group allocation and time. Models will further be adjusted for sex, primiparity, the baseline value of the outcome, stratum of health centers and interaction terms between these variables and time. Interactions between intervention allocation and baseline covariates will be inspected. The intervention effect will be statistically tested using a likelihood ratio test comparing a model with and without the interaction terms between intervention allocation and linear and nonlinear terms for time.

For the analysis of incidences (wasting) we will use mixed-effects Poisson regression models, while binary outcomes (eg. longitudinal prevalence of disease, stunting) will be analyzed using mixed-effects logistic regression models.

The applied mixed-effects models will use random effects for health center catchment area (level-3), village (level-2), individual (level-1) to take into account the effect of clustering at these levels and to estimate the standard error in an unbiased manner.

In order to assess the robustness of the findings, we will impute for missing data using a multiple imputation strategy.
Data management, data cleaning and statistical analyses will be conducted using Stata 13.1 (Statacorp, USA). The statistical significance for all tests will be set at 5% in case of testing main effects or 10% in case of interactions. All statistical tests will be two-sided.

4. Ethical approval

The study protocol will be submitted for revision to the IFPRI IRB (Washington DC, USA) and to the National Ethics Committee of Mali (Bamako, Mali). The intervention study will be registered at clinicaltrials.gov.

Care will be taken to provide the best available treatment to address children needs. In case the evaluator team would encounter children that suffer from acute malnutrition or from severe anemia (Hb<7 $g.dL^{-1}$), the mother-child pair will be referred with a letter of referral.

Following principles will be adopted:

- Upon Ethics committee approval of the protocol HKI Mali will inform local and regional health authorities and community representatives. In every village of the study area HKI representatives will present the study's objectives and procedures to village authorities (village leaders, religious leaders) and demand their authorization.

- Voluntary and informed participation: The study's objective and procedures will be explained to all caregivers and head of household of eligible children in their local language. All informants reserve the right to refuse to participate in the study. Each enumerator will be asked to read the consent statement in full form, slowly and in local language to the participant. They will then ask if the consent statement was understood and if there are any questions. Then the enumerator will ask the mother/caretaker if they would like to participate in the survey questions. At this time the informant will be given the opportunity to refuse and understand that they may also be able to refuse the study at any time during the survey with no repercussions to them or their family. Consent for children will be provided by their mothers or legal guardians. An informed consent form will be signed by the primary caregiver of the child and, if present, the head of household.
- Risks and serious adverse events: there are no known risks related to the evaluation methods (questionnaires, anthropometry). Good clinical practices will be respected by drawing capillary blood to dose hemoglobin concentration.
- Emergencies: All study subjects of both control and intervention arms will benefit from medical care in case a medical emergency is encountered during the evaluation visits. Medical emergencies include cases of severe anemia or any danger sign that would make competent medical staff conclude that hospitalization is required (eg. Non-response to induced pain, persisted vomiting, unconsciousness, etc.). The program will carry any medical costs related to such interventions (consultation, prescriptions, hospitalization if decided by competent medical staff) until discharge. Chronic diseases are not covered by this engagement. The management of acute malnutrition is available in every health center free of charge.
- Anonymity: Care will also be taken to ensure anonymity of participants during data collection, data management, data analysis and result dissemination. Data forms will be made anonymous by using identification codes.

5. Partnership and management

This study is a collaboration between HKI and IFPRI. HKI Mali country team under supervision of the HKI regional office will be the implementer of the PROMIS program. IFPRI is in charge of the evaluation of the study. Both organizations guarantee the adherence to the study protocol. Table 3 enlists the responsibilities of all actors of the project. In table 3 an overview of all involved parties is provided.

Table 3 Overview of project's actors and stakeholders

Project stakeholder	Name	Role
Sponsor and Scientific Partner	**IFPRI**<br>2033 K St, NW<br>Washington, DC 20006-1002<br>USA	- Principal investigator<br>- Design of the of study

	Phone: +1 202-862-5600<br>Fax: +1 202-467-4439<br>Email:<br>- Marie Ruel (PI)<br>M.Ruel@cgiar.org<br>- Lieven Huybregts<br>L.Huybregts@cgiar.org	- Quality control of the data collection<br>- Dissemination of the results
	**IFPRI Dakar**<br>Titre 3396, Lot #2<br>BP 24063 Dakar Almadies<br>Senegal<br>Phone: +221.33.869.9800<br>Email:<br>- Elodie Becquey<br>E.Becquey@cgiar.org<br>- Agnes Le Port<br>A.Leport@cgiar.org<br>- Rahul Rawat<br>R.Rawat@cgiar.org	- Guarantor of research protocol
**Implementing Agency**	**HKI West Africa**<br>28, Nord Liberté 6. VDN X Route du Front de Terre à Dakar. N° 1A. BP. 16<br>511 Dakar Fann, Senegal<br>Telephone: + 00 221 33 869 55 01 Fax: + 00 221 33 827 24 06<br><br>- Raphael Bajay Tchumah (Program manager)<br>RTchumah@hki.org<br>- Jennifer Nielsen<br>jnielsen@hki.org	- Coordination of implementation of program<br>- Project management<br>- Training and supervision CHW, CHV and mother groups<br>- Financial management<br>- Ensure logistics of SQ-LNS
	**HKI Mali**<br>BP E1557. Hamdallaye ACI 2000, Rue 339, Porte 118 Bamako, Mali<br>Telephone: +223 20 21 08 21<br>- Marily Knieriemen<br>mknieriemen@hki.org<br>- Lazare Coulibaly<br>lcoulibaly@hki.org<br>- Abdoul Salam Savadogo<br>asavadogo@hki.org	

6. Budget

The PROMIS project is funded by the Department of Foreign Affairs, Commerce and Development of Canada. Table 4 shows the main budget lines.

Table 4: Overview Project budget

**Type**	**Cost (US$)**
Materials	
Service Contracts	
Reimbursement of medical costs	
Incidental (10%)	
Overhead (20%)	
**Totals**	

7. TIMELINE

In Table 5 an overview of the different components of the PROMIS program is given. The implementation phase of the study will end early 2017. Data analysis and dissemination of findings in scientific journals and technical reports on partners' websites will happen in 2017.

Table 5: Timeline of the PROMIS program

Activities	2014		2015				2016				2017	
	Q3	Q4	Q1	Q2	Q3	Q4	Q1	Q2	Q3	Q4	Q1	Q2
Obtaining Ethics approval		X										
Training enumerators		X										
Baseline survey		X										
Refresher training CHV, CHV, mother groups		X										
Longitudinal survey			X	X	X	X	X	X	X			
Endline survey										X		
Data analysis											X	
Dissemination of results											X	

8. Reference List

1. Black RE, Victora CG, Walker SP, et al. Maternal and child undernutrition and overweight in low-income and middle-income countries. *Lancet*. 2013;382(9890):427-451.

2. WHO. *Technical Note: Supplementary Foods for the Management of Moderate Acute Malnutrition in Infants and Children 6–59 Months of Age*.; 2012:1-12. doi:10.1227/00006123-199112000-00028.

3. Nyirandutiye DH, Ag Iknane A, Fofana A, Brown KH. Screening for acute childhood malnutrition during the National Nutrition Week in mali increases treatment referrals. *PLoS One*. 2011;6(6):e14818. doi:10.1371/journal.pone.0014818.

4. Bhutta ZA, Das JK, Rizvi A, et al. Evidence-based interventions for improvement of maternal and child nutrition: What can be done and at what cost? *Lancet*. 2013;382(9890):452-477.

5. De-Regil LM, Suchdev PS, Vist GE, Walleser S, Peña-Rosas JP. Home fortification of foods with multiple micronutrient powders for health and nutrition in children under two years of age (Review). *Evid Based Child Health*. 2013;8(1):112-201. doi:10.1002/ebch.1895.

6. Bhutta Z, Rivzi A, Raza F, et al. A comparative evaluation of multiple micrnonutrient and iron-folic acid supplementation during pregnancy in Pakistan: impact on pregnancy outcomes. *Food Nutr Bull*. 2009;(30):S496-S505.

7. Huybregts L, Houngbé F, Salpéteur C, et al. The effect of adding ready-to-use supplementary food to a general food distribution on child nutritional status and morbidity: a cluster-randomized controlled trial. *PLoS Med*. 2012;9(9):e1001313. doi:10.1371/journal.pmed.1001313.

8. Iannotti LL, Jean S, Dulience L, et al. Linear growth increased in young children in an urban slum of Haiti : a randomized controlled trial of a lipid-based nutrient supplement 1 – 3. 2014:198-208. doi:10.3945/ajcn.113.063883.198.

9. Dewey KG, Yang Z, Boy E. Systematic review and meta-analysis of home fortification of complementary foods. *Matern Child Nutr*. 2009;5:283-321. doi:10.1111/j.1740-8709.2009.00190.x.

10. Lewin S, Munabi-Babigumira S, Glenton C, et al. Lay health workers in primary and community health care for maternal and child health and the management of infectious diseases: a review synopsis. *Cochrane Database Syst Rev*. 2010;28(3):243-5. doi:10.1111/j.1525-1446.2011.00950.x.

11. Macro ORC. *Enquête Démographique et de Santé Du Mali EDSM-V Rapport Préliminaire*.; 2013.

12. Bernabé BP. *Community Based Management of Acute Malnutrition in Koutiala District: A Coverage Assessment*.; 2013:50.

13. Victora CG, de Onis M, Hallal PC, Blössner M, Shrimpton R. Worldwide timing of growth faltering: revisiting implications for interventions. *Pediatrics*. 2010;125(3):e473-80. doi:10.1542/peds.2009-1519.

14. Hayes RJ, Bennett S. Simple sample size calculation for cluster-randomized trials. 1999:319-326.

15. Schmidt W-P, Genser B, Barreto ML, et al. Sampling strategies to measure the prevalence of common recurrent infections in longitudinal studies. *Emerg Themes Epidemiol*. 2010;7(1):5. doi:10.1186/1742-7622-7-5.

16. Weinstein MC, Siegel JE, Gold MR, Kamlet MS, Russell LB. Recommendations of the Panel on Cost-effectiveness... [JAMA. 1996] - PubMed result. *JAMA*. 1996;276(15):1253-8. Available at: http://www.ncbi.nlm.nih.gov/pubmed/8849754.

17. Tan-Torres Edejer T, Baltussen R, Adam T, Hutubessy R, Acharya A, Evans DB and Murray C. WHO GUIDE TO COST-EFFECTIVENESS ANALYSIS. In: *Global Programme on Evidence for Health Policy ,World Health Organization, Geneva*.; 2003.

Annex 1: Description of the SQ-LNS

SQ-LNS (Nutriset, France) is a fortified spread that consists of peanut paste, milk powder, vegetable oil and maltodextrin. This food matrix is fortified with a premix of multiple micronutrients (Table A). The product is packaged in daily portions of 20g. This supplement has previously been used in several programs in Sub-Saharan Africa for the prevention of child undernutrition.
Upon distribution, CHW or CHV will explain the benefitting mother that the product serves primarily as a home-fortification and should not be a substitute for breastfeeding or for habitual complementary foods. It will be recommended that caregivers offer the supplement after breastfeeding is completed. The supplement can either be consumed as such, or can be mixed with the complementary food.

Table A: Nutritional composition of SQ-LNS

	**For 20 g (recommended daily dose)**		**For 20 g (recommended daily dose)**
Energy	118 kcal	Vitamin A	0.4 mg
Proteins	2.6 g	Vitamin B1	0.3 mg
Lipids	9.6 g	Vitamin B2	0.4 mg
Calcium	280 mg	Niacin	4.0 mg
Phosphorus	190 mg	Pantothenic acid	1.8 mg
Potassium	200 mg	Vitamin B6	0.3 mg
Magnesium	40 mg	Folic acid	80 µg
Zinc	8 mg	Vitamin B12	0.5 µg
Copper	0.34 mg	Vitamin C	30 mg
Iron	6 mg	Vitamin D	5 µg
Iodine	90 µg	Vitamin E	6.0 mg
Selenium	20 µg	Vitamin K	30 µg
Manganese	1.2 mg		

Note: 20 g of LNS Infant provide a minimum of 4.46 g LA (Linoleic Acid) and a minimum of 0.42 g ALA (α-Linolenic Acid).
