# Supplementary material for: Impact on child acute malnutrition of integrating small-quantity lipid-based nutrient supplements into community-level screening for acute malnutrition: A cluster-randomized controlled trial in Mali
Source: PLoS Med. 2019 Aug 27;16(8):e1002892. doi: 10.1371/journal.pmed.1002892 (PMC6711497; doi:10.1371/journal.pmed.1002892)
Supplement: S2 Text — PROMIS, Innovative Approaches for the Prevention of Childhood Malnutrition. (DOCX) [file pmed.1002892.s010.docx]

**Data analysis plan for:**

PROMIS Mali- Impact on child acute malnutrition of integrating small-quantity lipid-based nutrient supplements into community-level screening for child acute malnutrition: A cluster randomized controlled trial in Mali

## Primary study outcomes

### *Repeated cross-sectional study*

Primary study outcomes for the cross-sectional study design are:

1. the prevalence of AM defined by a WLZ<-2 or a MUAC< 125mm (only in children older than 6 months) or the presence of bilateral pitting edema;
2. AM screening coverage defined as the number of children screened in the month preceding the survey (as reported by the caregiver) over the total number of eligible study children;
3. AM treatment compliance defined as the number of AM children under appropriate treatment at the time of the survey over the total number of AM cases identified in the study sample.

### *Longitudinal study*

For the longitudinal study, primary outcomes are:

1. Incidence of AM (same definition of AM as above);
2. monthly AM screening coverage (the number of children screened each month over the total number of eligible study children);
3. AM treatment compliance (the number of AM children adhering to weekly or bi-weekly treatment until discharged over the total number of AM children that are scheduled for treatment).

## Secondary study outcomes

Secondary study outcomes for the cross-sectional study are:

- Mean HAZ-score at endline (using WHO 2006 growth reference)

- Mean height at endline;

- Mean WHZ-score at endline (using WHO 2006 growth reference);

- Mean MUAC at endline;

- Mean hemoglobin concentration at endline;

- Prevalence of child anemia (Hb concentration<11g.dL-1) at endline;

- Prevalence of child severe anemia (Hb concentration<7g.dL-1) at endline;

- Prevalence of child stunting defined by HAZ<-2 (using WHO 2006 growth reference).

- Prevalence of MAM defined by a -3≤WHZ<-2 (using WHO 2006 growth reference) or a 115mm≤MUAC<125mm

- Prevalence of SAM defined by a WHZ<-3 (using WHO 2006 growth reference)or bilateral pitting edema or a MUAC<115mm;

- Prevalence of severe stunting defined by a HAZ<-3 (using WHO 2006 growth reference);

- Caregiver’s knowledge of indicators of WHO recommended IYCF, CMAM and WASH;

- Caregiver’s practices related to IYCF, ENA and WASH;

- Immunization coverage (defined as the recommended immunization contacts by child age at which these vaccines should be received, following the national guidelines in Mali allowing for a tolerance 4 weeks after the recommended age point).
- Weight-for-age Z-score (added on June 20, 2019)
- Prevalence of underweight (added on June 20, 2019)

Secondary study outcomes for the longitudinal study are:

- Incidence of child stunting defined by HAZ<-2 (using WHO 2006 growth reference) in children followed-up monthly from 6 to 23 months of age;
- Longitudinal prevalence of AM, MAM and SAM defined by time the child is AM, MAM, SAM over the total follow-up time respectively;
- Change in mean AM prevalence over time
- Treatment compliance of MAM and SAM (the number of MAM or SAM children adhering to weekly or bi-weekly treatment until discharged over the total number of MAM or SAM children that are scheduled for treatment).
- Enrollment into CMAM of AM, MAM or SAM children (caregiver report)
- Recovery rates of AM, MAM or SAM (defined as the proportion of children who are free from AM for at least one monthly measurement over the total number of children suffering from AM, MAM, and SAM respectively);
- Relapse rate after successful treatment of AM (%WHZ<-2 or MUAC<125mm or bilateral pitting edema after discharge from MAM or SAM treatment program);
- Mean episode length for AM (an episode of AM is defined as starting from the moment a child is found to be acutely malnourished at the monthly survey visit until the moment the child is free from AM for at least one monthly measurement);
- Mean episode length for MAM or SAM (a MAM or SAM episode is defined as starting from the moment a child is found to be MAM or SAM at the monthly survey visit until the moment the child is free from AM for at least one monthly measurement);
- Linear growth velocity (HAZ increment/month);
- Ponderal growth velocity (WHZ increment/month);
- Weight gain (weight increment/month);
  - MUAC gain (MUAC increment/month);
- Longitudinal prevalence of Infant morbidity : acute respiratory infections, fever, diarrhea, vomiting and malaria.
- Change in IYCF practices and caregiver knowledge.
- Immunization coverage (defined as the recommended immunization contacts by child age at which these vaccines should be received, following the national guidelines in Mali allowing for a tolerance 4 weeks after the recommended age point).

## Sample size calculation for primary study outcomes

We used Hayes and Bennet’s formulas^[[1]](#footnote-1)^ to calculate the necessary sample sizes for the repeated cross-sectional and the longitudinal study.

### *Cross-sectional study*

### Assuming a coefficient of inter-cluster (i.e. between health center catchment areas) variation k of 0.25, a non-response rate of 15%, a type I error of 5% and a statistical power of 80%, we calculate that with an average cluster (i.e. HC catchment area) size of 48 children, 48 clusters (i.e. an overall sample size of 2,304 children) are needed for each survey round to detect a decrease in the prevalence of AM of 5.3 percentage points assuming a baseline prevalence of 18.0% [24]. This sample size allows detecting a difference in AM screening coverage of 6.7 percentage points and a difference in AM treatment coverage of 14.7 percentage points between study arms assuming baseline values of 25% for both outcomes.

### *Longitudinal study*

Assuming a coefficient of inter-cluster variation k of 0.2, a dropout rate of 20%, a type I error of 5% and a statistical power of 80%, we needed to recruit 24 children in each of the 48 clusters (i.e. an overall sample size of 1,152 children total) to detect a 23.5% reduction in the incidence of AM over 18 months of follow-up, assuming a baseline incidence of 0.61 case^[[2]](#footnote-2)^ per child-year. This sample size allows us to detect a difference in AM screening coverage of 4.9 percentage points and a difference in treatment coverage of 9.6 percentage points during the 18 month of follow-up assuming an incidence of 0.61 cases per child-year and baseline values of 25% for both outcomes.

# Randomization procedure

We apply a stratified random allocation of the HC catchment areas to control and intervention study groups. Health centers are governed by their own community health association and operate autonomously. They are thus expected to be quite heterogeneous in terms of organization and performance. Stratifying clusters prior to randomization ensures a more balanced distribution of cluster-level covariates between study arms. We first stratify the health centers by hierarchical clustering using a set of criteria:

- Type of staff working in HC

- Accessibility during rainy season

- Type of the catchment area (urban/semi-urban/rural)

- Number of villages covered

- Number of villages with community health workers (CHW)

- Vaccination coverage

- Total number of children 6-23.9 months

- Proportion MAM admissions/total population

- Proportion SAM admissions/total population

- Distance between villages and HC

- Distance between HC and District hospital

Based on the visual inspection of the obtained cluster dendrogram obtained from hierarchical clustering with complete linkage, we subdivide the clusters into strata for the Bla and San district. Random allocation to control or intervention groups is conducted within each stratum and health district during a community ceremony in Bla and San in the presence of local health authorities.

During a public lottery ceremony, forty-eight identical pieces of paper with either ‘control’ (n = 24) or ‘intervention’ (n = 24) written on them are mixed in a bag by the project coordinator of HKI. Per stratum and health district the bag contains the same number of control and intervention pieces of paper. Per stratum and health district, the concerning directors of the health centers draw one piece of paper, thus allocating his HC catchment area to the control or intervention study group.

# Sampling

A census to identify infants will be organized prior to the start of the cross-sectional surveys and prior to the beginning of the longitudinal study. We exclude villages and small settlements with a population of less than 300 to lower the logistical cost. Since age is an important predictor of AM and nutrition and health-related practices, we stratify children from the census list in three equal age groups (6–11 months; 12–17 months; 18–23 months ) and draw a random sample from each age group for the cross-sectional study.

## Statistical analysis

Data will be analyzed on intent-to-treat basis. To allow for an analysis “as randomized” in the presence of missing data, we will impute missing data of the longitudinal study using an appropriate multiple imputation strategy under the *missing at random* assumption. Data management, data cleaning, and statistical analyses will be done using Stata 14 or 15 (Statacorp, USA). The statistical significance will be set at 5%. All statistical tests will be two-sided. The analysis of primary study outcomes will be adjusted for multiple testing (n=6 primary study outcomes).

Any imbalance in covariates at baseline will be assessed in absolute terms (e.g. is the difference in means or prevalence larger than 5%?). Baseline differences will not be subject to statistical testing.

### *Repeated cross-sectional study*

The repeated cross-sectional study design will be used to estimate program impact after 2 years of program implementation. Linear and linear probability mixed-effect regression models will be used for continuous and binary outcomes respectively. In case of linear probability models, we will use a robust estimation of standard errors to account for heteroscedasticity of the residuals (added on June 20, 2019). Although the randomization is expected to minimize average differences at baseline between groups, we will adjust regression models for baseline values of the outcome of interest and covariates that should not have changed as a consequence of the intervention to improve the precision of the estimates. Regression models will further be adjusted for clustering at HC catchment area level. Health center sampling stratum and district will be added as a fixed effects in the regression models. Exploratory analysis will assess effect modification by HC and CHW characteristics by testing interaction terms and if statistically significant at 10% level considered for sub-group analysis.

### *Longitudinal study*

Ponderal (weight, MUAC and WHZ increments), linear growth (length and HAZ increments), screening and treatment coverage obtained from the longitudinal study will be modeled using mixed-effects model with restricted splines to account for a likely non-linear character. For binary outcomes, we will use linear probability spline models with robust estimation of standard errors (added on June 20, 2019). Covariates include the intervention group allocation, spline terms for time (in months), and interaction terms between intervention group allocation and spline terms time. Models will further be adjusted for sex, first live birth (yes/no), the baseline value of the outcome, stratum of health centers and district. The intervention effect will be statistically tested using a likelihood ratio test (or by an equivalent omnibus test) comparing a model with and without the interaction terms between intervention allocation and spline terms for time. Interactions between intervention allocation and baseline covariates will be inspected to assess possible effect modification.

For the analysis of AM incidence and the longitudinal prevalence of morbidity, we will use mixed-effects Poisson regression models with robust estimation of standard errors. The mixed-effects models will be adjusted for clustering by HC catchment area and individual to estimate the correct standard errors. Models will be further adjusted for child age, first live born (yes/no), child sex, health center sampling stratum and district.

1. Hayes RJ, Bennett S. (1999) Simple sample size calculation for cluster-randomized trials. International Journal of Epidemiology; 319–26. [↑](#footnote-ref-1)
2. We estimated a baseline AM incidence of 0.61 cases per child-year assuming a 18% AM prevalence (~16% MAM prevalence) and an incidence conversion factor K of 3.2 (Isanaka et al. 2011) [↑](#footnote-ref-2)
